# CADM1 is essential for KSHV-encoded vGPCR-and vFLIP-mediated chronic NF-κB activation

**DOI:** 10.1371/journal.ppat.1006968

**Published:** 2018-04-26

**Authors:** Richard Hunte, Patricia Alonso, Remy Thomas, Cassandra Alexandria Bazile, Juan Carlos Ramos, Louise van der Weyden, Juan Dominguez-Bendala, Wasif Noor Khan, Noula Shembade

**Affiliations:** 1 Department of Microbiology and Immunology, Viral Oncology Program, Sylvester Comprehensive Cancer Center, Miller School of Medicine, The University of Miami, Miami, FL, United States of America; 2 Qatar Biomedical Research Institute, Doha, Qatar; 3 Department of Medicine, Division of Hematology-Oncology, Sylvester Comprehensive Cancer Center, and Center for AIDS Research and Department of Microbiology and Immunology, University of Miami Miller School of Medicine, Miami, Florida, United States of America; 4 Wellcome Trust Sanger Institute, Wellcome Trust Genome Campus, Hinxton, Cambridge, United Kingdom; 5 Diabetes Research Institute, Miller School of Medicine, The University of Miami, Miami, FL, United States of America; University of Southern California, UNITED STATES

## Abstract

Approximately 12% of all human cancers worldwide are caused by infections with oncogenic viruses. Kaposi's sarcoma herpesvirus/human herpesvirus 8 (KSHV/HHV8) is one of the oncogenic viruses responsible for human cancers, including Kaposi’s sarcoma (KS), Primary Effusion Lymphoma (PEL), and the lymphoproliferative disorder multicentric Castleman’s disease (MCD). Chronic inflammation mediated by KSHV infection plays a decisive role in the development and survival of these cancers. NF-κB, a family of transcription factors regulating inflammation, cell survival, and proliferation, is persistently activated in KSHV-infected cells. The KSHV latent and lytic expressing oncogenes involved in NF-κB activation are vFLIP/K13 and vGPCR, respectively. However, the mechanisms by which NF-κB is activated by vFLIP and vGPCR are poorly understood. In this study, we have found that a host molecule, Cell Adhesion Molecule 1 (CADM1), is robustly upregulated in KSHV-infected PBMCs and KSHV-associated PEL cells. Further investigation determined that both vFLIP and vGPCR interacted with CADM1. The PDZ binding motif localized at the carboxyl terminus of CADM1 is essential for both vGPCR and vFLIP to maintain chronic NF-κB activation. Membrane lipid raft associated CADM1 interaction with vFLIP is critical for the initiation of IKK kinase complex and NF-κB activation in the PEL cells. In addition, CADM1 played essential roles in the survival of KSHV-associated PEL cells. These data indicate that CADM1 plays key roles in the activation of NF-κB pathways during latent and lytic phases of the KSHV life cycle and the survival of KSHV-infected cells.

## Author summary

Infection with Kaposi sarcoma herpesvirus (KSHV) leads to the development of Kaposi sarcoma (KS), primary effusion lymphoma (PEL), multicentric Castleman disease (MCD), and AIDS-associated Kaposi's sarcoma (AIDS-KS). KSHV infection exhibits both latent and lytic phases with distinct viral gene expression. Chronic inflammation mediated by a transcription factor, NF-κB, during KSHV latent and lytic phases play critical roles in the development of KSHV-associated malignancies. One of the main mechanisms of persistent NF-κB activation in KSHV-infected cells is dysregulation of host gene expression and their normal functions by viral oncogenes. However, the cellular host factors and the mechanisms required for persistent NF-κB activation in KSHV-infected cells remains largely unknown. Here, we focused on host tumor suppressor Cell adhesion molecule 1 (CADM1) that is robustly upregulated within a few hours of KSHV infection. Although the role of CADM1 as a tumor suppressor in solid tumors is partially known, its role in oncogenic virus-mediated tumors is poorly understood. We characterized the role of CADM1 in persistent NF-κB activation in KSHV infected cells. We found that CADM1 is required for both latent and lytic, vFLIP and vGPCR, oncogenes of KSHV to maintain chronic NF-κB activation. We further demonstrated that CADM1 is critical for the survival of KSHV-associated PEL cells. Our study reveals a novel mechanism of chronic NF-κB activation by CADM1 in KSHV-infected cells.

## Introduction

Kaposi’s sarcoma herpesvirus/Human herpesvirus 8 (KSHV/HHV8) is a latent, double-stranded DNA γ-2 herpesvirus that exhibits a tropism for peripheral blood B cells, endothelial cells, monocytes, and epithelial cells [1–3]. KSHV is the etiological agent of Kaposi sarcoma (KS), primary effusion lymphoma (PEL) and the lymphoproliferative disorder multicentric Castleman’s disease (MCD) [4, 5]. KS has been classified into four different subtypes: classic, which is prevalent in elderly men of Mediterranean or Eastern European Jewish ancestry; endemic, which is widespread in Central and Eastern Africa; iatrogenic, a subtype that inflicts immunosuppressed patients; and epidemic or AIDS-associated KS [5, 6]. KSHV-induced KS and B cell lymphomas are increased dramatically in human immunodeficiency virus 1 (HIV-1)-infected individuals receiving anti-retroviral therapy (ART) [7, 8]. Chronic inflammatory cytokines are increased significantly in AIDS/KS patients, and ensures the reactivation and infection of KSHV [7, 9–11]. The chronic inflammatory condition in AIDS/KS patients is maintained primarily by aberrant activation of NF-κB, a family of transcription factors regulating inflammation and cell survival [12, 13]. Furthermore, chronic activation of NF-κB is critical for the survival of PEL cells [14–18].

NF-κB plays critical roles in the induction of genes that are involved in cell growth, differentiation, activation, and survival [19]. In the canonical NF-κB pathway, NF-κB dimers are sequestered in the cytoplasm as latent transcription factors by inhibitory molecules termed IκBs [19, 20]. In response to proinflammatory cytokines, pathogen infections, or antigens the canonical NF-κB pathway is activated in membrane lipid rafts resulting in the phosphorylation, ubiquitination, and proteasomal degradation of IκBα [19]. Phosphorylation of IκBα is mediated by a large multi-subunit kinase complex consisting of the catalytic subunits IKKα and IKKβ and a regulatory subunit, IKKγ (also known as NEMO) [19]. The IKK complex is regulated by multiple post-translational modifications including phosphorylation and ubiquitination. Upon receptor stimulation key signaling molecules undergo K63-linked polyubiquitination, which is critical for the activation of the IKK complex [21]. In the non-canonical NF-κB pathway, p100 is processed to p52 upon phosphorylation by IKKα, which is activated by NF-κB inducing kinase (NIK), upon stimulation of receptors of the TNF superfamily including BAFF-R and lymphotoxin β receptor (LTβR) [22]. Viral oncogenes, such as Tax of Human T-cell leukemia virus type 1 (HTLV-1), LMP-1 of Epstein-Barr Virus (EBV) and vFLIP of KSHV constitutively induce p100 processing and generation of p52 [23–25].

The KSHV genome encodes multiple proteins that can mediate chronic activation of NF-κB, including the homologue of FADD-like interleukin (IL)-1b-converting enzyme (FLICE/caspase-8) inhibitory protein (vFLIP), which mediates chronic activation of NF-κB. Similar to HTLV-1 Tax, vFLIP interaction with IKKγ leads to the activation of NF-κB pathways in KSHV-infected cells [15, 26, 27]. However, it is unclear whether vFLIP also uses distinct mechanisms of NF-κB activation than that used by HTLV-1 Tax. Another NF-κB-activating protein is the constitutively active KSHV-encoded oncogene viral G-protein-coupled receptor (vGPCR, open reading frame 74), with close homology to mammalian chemokine receptors CXCR1 and CXCR2. Forced expression of vGPCR in endothelial cells, or in mice infected with recombinant murine gamma herpesvirus carrying KSHV vGPCR, or as a transgene in mice is sufficient to induce angiogenic KS-like tumors [28–31]. vGPCR constitutively activates MAPKs, NF-κB, NFAT, and PI3K-AKT-mTOR pathways, which are critical for the development of angio-proliferative tumors [16, 32–37]. Interestingly, recent studies have demonstrated that vGPCR maintains chronic activation of NF-κB in PEL cell lines and primary B cells derived from KS patients [16]. The C-terminal YGLF motif of vGPCR mediates interaction with AP2 and is critical for vGPCR localization between the plasma membrane and clathrin-coated vesicles [16]. Mutating the YGFL motif of vGPCR or disrupting the AP2 complex restricts vGPCR localization to the plasma membrane and leads to massive and chronic activation of NF-κB. However, the molecular mechanisms of vGPCR-mediated NF-κB activation remain poorly understood.

It remains unclear exactly how vFLIP and vGPCR promote the catalytic activity of the IKK kinase complex. Typically, IKK and NF-κB activation is initiated in the membrane microdomains, known as lipid rafts, by the BCR in B cells and by the TCR in T cells [38–40]. The membrane lipid rafts (10–200 nm in diameter) are heterogeneous, highly dynamic subdomains of the plasma membrane composed of glycosphingolipid- and cholesterol-enriched, detergent-resistant microdomains, which play critical roles in signal transduction[41, 42]. Although membrane lipid rafts serve as scaffolds for several signaling receptors to communicate extracellular stimuli to the intracellular milieu, recent studies suggest that lipid rafts are also crucial for intracellular viral oncogenes to transmit the signal downstream. For example, the viral oncogene Tax of HTLV-1 and LMP-1 of EBV initiate NF-κB activation in the membrane lipid rafts [43–46].

Cell adhesion molecule 1 (CADM1; also known as RA175/SynCAM1/TSLC1/ Necl-2/ IGSF4), is a member of the immunoglobulin superfamily (IgSF) and serves critical roles in the adhesion of spermatogenic cells to Sertoli cells [47, 48]. CADM1 is highly upregulated and serves as a marker of HTLV-1-infected adult T-cell leukemia (ATL) cells [49]. Recent studies have demonstrated that the upregulation of CADM1 may also contribute to obesity, although the mechanism is not fully understood [50]. Previous studies have shown that CADM1 is expressed in the cytoplasm and cell membrane where it exerts its signaling functions [44, 51–53]. It has also been demonstrated that CADM1 plays unique roles in T-cell signaling and activation [54]. Our previous findings have demonstrated that the HTLV-1-encoded oncogene, Tax, required CADM1 for chronic NF-κB activation in membrane lipid rafts [44]. CADM1 functions as a critical adaptor molecule for K63-linked polyubiquitination of Tax in the membrane lipid rafts [44]. These results suggest that CADM1 is a key molecule for HTLV-1-mediated tumorigenesis. However, it remains unclear if CADM1 promotes oncogenesis by other viruses, such as KSHV.

In this study, we have demonstrated that the mRNA and protein expression of CADM1 were robustly upregulated in KSHV-infected human primary B cells and PEL cell lines. Further investigation determined that KSHV-vFLIP and vGPCR interacted with CADM1 to maintain the persistent activation of NF-κB. Additionally, the PDZ binding motif localized at the carboxyl terminus of CADM1 is essential for both vFLIP and vGPCR to maintain chronic NF-κB activation. vFLIP is localized to membrane lipid rafts together with CADM1 and initiated NF-κB activation in PEL cells. In addition, CADM1 played essential roles in the survival of KSHV-associated PEL cells. These data indicate that CADM1 plays key roles in the activation of NF-κB during the latent and lytic phases of the KSHV life cycle and the survival of KSHV-infected cells.

## Results

### The expression of CADM1 is upregulated in KSHV-infected cells

**Fig 1:**
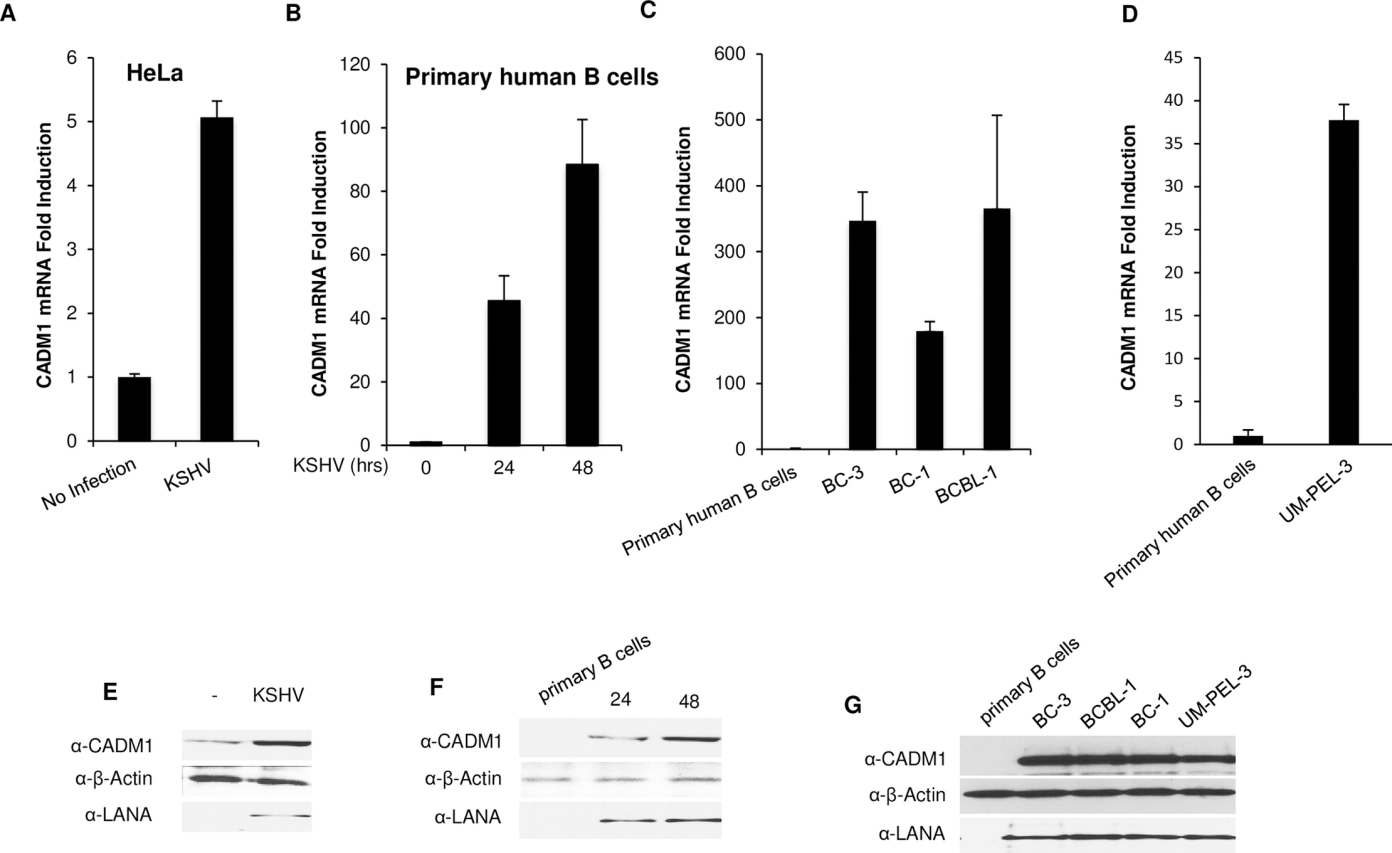
CADM1 expression is upregulated in KSHV-infected cells. (A-B) Quantitative real-time PCR (qRT-PCR) analysis of CADM1 from HeLa and primary human B cells with and without KSHV infection. (C-D) qRT-PCR analysis of CADM1 from KSHV-associated PEL cell lines (BC-1, BC-3, BCBL-1, and UM-PEL-3) (error bars, s.e.m. of triplicate samples). (E-G) Western blot analysis of CADM1 and LANA in KSHV-infected HeLa, human primary B cells, and PEL cell lines (BC-1, BC-3, BCBL-1, and UM-PEL-3).

Although CADM1 functions as a tumor suppressor in non-small cell lung cancer (NSCLC) and is downregulated in many solid tumors, its expression is significantly upregulated in the context of infection with the oncogenic retrovirus HTLV-1, and associated ATL tumor cells. The expression of CADM1 is essential for the proliferation and survival of HTLV-1-transformed T cells and ATL cells through activation of NF-κB [44, 49, 55]. However, it is unknown if the expression of CADM1 is also upregulated in KSHV-infected cells and KSHV-associated PEL cells. To examine CADM1 expression in KSHV-infected cells, we first infected HeLa cells, human umbilical vein endothelial cells (HUVEC), and primary human B-cells isolated from peripheral blood mononuclear cells (PBMCs) from healthy volunteers with KSHV at 0.1 multiplicity of infection (MOI) as described previously [56]. Protein lysates and mRNAs were prepared post-infection. We examined CADM1 mRNA levels by quantitative real-time PCR (qRT-PCR) and protein expression by Western blot in the absence or presence of KSHV infection. The KSHV-associated latency-associated nuclear antigen (LANA) protein expression was used to confirm KSHV infection. Surprisingly, CADM1 mRNA and protein expression were significantly upregulated in KSHV-infected HeLa, HUVEC, and primary human B-cells compared to uninfected cells (Figs 1A, 1B, 1E, 1F and S1). As expected, LANA protein expression was detected only in the KSHV-infected cells (Figs 1E, 1F, 1G and S1). We next determined if CADM1 mRNA and protein expression were upregulated in primary effusion lymphoma (PEL) cell lines (BC-1, BC-3, BCBL-1, and UM-PEL-3) that are latently infected with KSHV. Indeed, we found that CADM1 mRNA and protein expression were upregulated in all the PEL (BC-1, BC-3, BCBL-1, and UM-PEL-3) cell lines, as detected by qRT-PCR and Western blotting, compared to uninfected primary human B-cells (Fig 1C, 1D and 1G). We also determined CADM1 protein expression in primary human B-cells, PEL (BC-1, BC-3, BCBL-1, and UM-PEL-3), and non-infected BJAB cell lines by flow cytometry. We found that CADM1 protein expression was upregulated in all the PEL (BC-1, BC-3, BCBL-1, and UM-PEL-3), and non-infected BJAB cell lines compared to uninfected primary human B-cells (Fig 1E, 1F and 1G and S2A–S2F Fig). Collectively, these results indicate that KSHV infection is associated with the upregulation of CADM1. Previous studies have shown that CADM1 expression is upregulated in response to NF-κB activation [44]. Therefore, we examined whether CADM1 expression can also be induced by the NF-κB inducers and KSHV oncogenes, vGPCR and vFLIP. HeLa cells were transfected with vGPCR or vFLIP and mRNAs were subjected to qRT-PCR. We found that CADM1 mRNA expression was upregulated in both vGPCR and vFLIP expressing HeLa cells (S3 Fig).

### CADM1 is required for KSHV-encoded vFLIP to activate NF-κB

**Fig 2:**
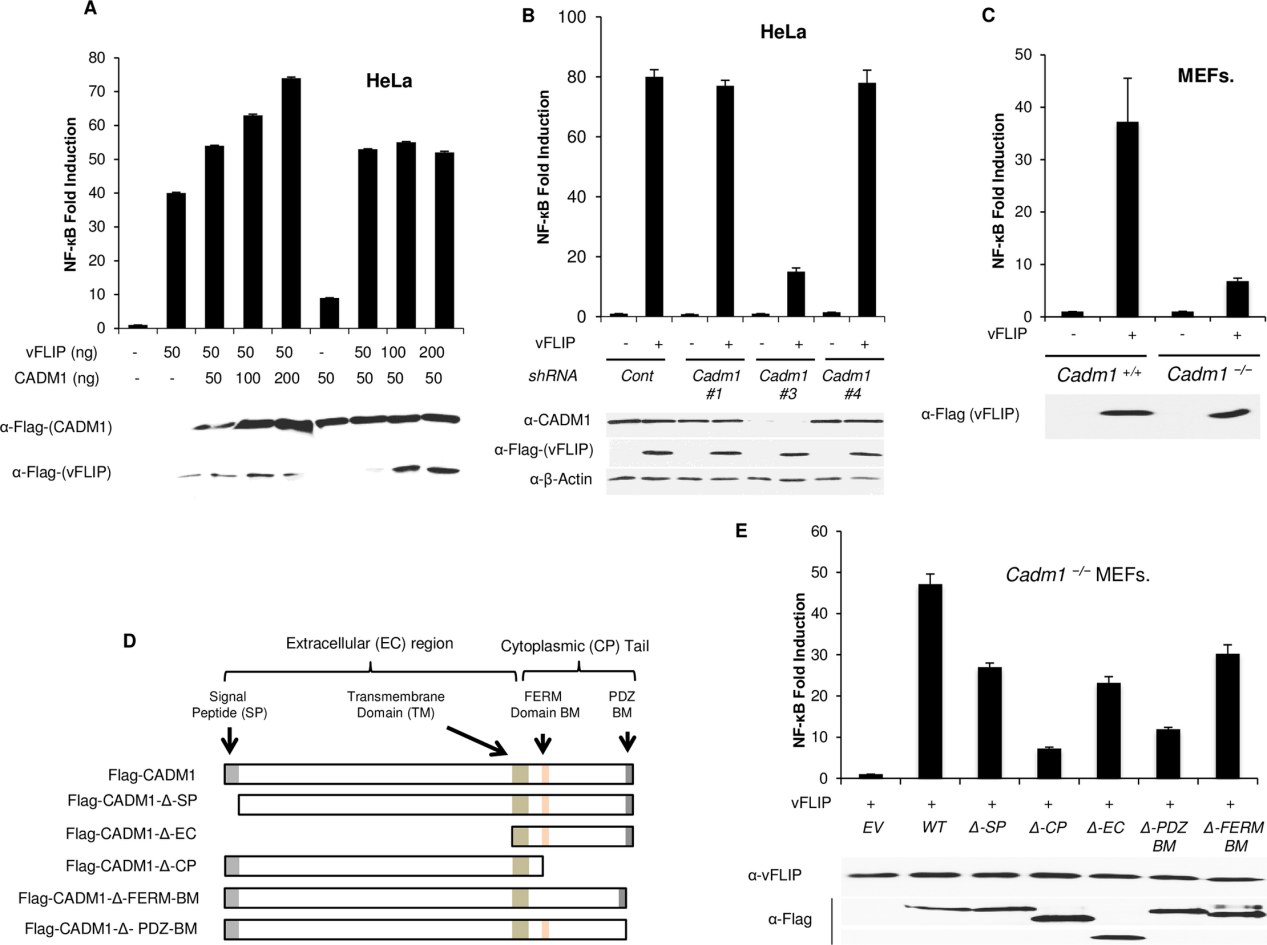
CADM1 is required for KSHV vFLIP to activate NF-κB. (A) NF-κB luciferase assay using lysates of HeLa cells expressing increasing amounts of CADM1 or vFLIP. HeLa cells were transfected with increasing amounts of either CADM1 or vFLIP with κB‐TATA Luc and pRL‐tk plasmids. After 36 hours, lysates were subjected to dual luciferase assays. The lysates were also subjected to immunoblotting to examine CADM1 and vFLIP expression using anti-Flag antibody. (B) NF-κB luciferase assay using lysates of HeLa cells stably expressing control scrambled shRNA or three different CADM1 shRNAs and transfected with pRL-tk, κB-TATA Luc and vFLIP as indicated. Immunoblot analyses of CADM1 protein expression in HeLa cells after transduction with lentiviruses expressing different shRNAs targeting distinct sequences of the CADM1 transcript. (C) NF-κB luciferase assay using lysates of *Cadm1*^*+/+*^ and *Cadm1*^*−/−*^ MEFs transfected with pRL-tk internal control Renilla luciferase plasmid, κB-TATA Luc and vFLIP as indicated. The lysates were also subjected to immunoblotting to examine vFLIP expression. (D) A schematic overview of the FLAG-CADM1 deletion mutants ΔSP, ΔCP, ΔEC, ΔPDZ-BM and ΔFERM. (E) NF-κB luciferase assay of lysates of *Cadm1*^*−/−*^ MEFs transfected with an NF-κB firefly luciferase reporter and a renilla luciferase vector reporter together with empty vector or an expression vector for Flag-tagged wild-type CADM1, CADM1 ΔSP, CADM1 ΔCP, CADM1 ΔEC, CADM1 ΔPDZ-BM and CADM1 ΔFERM-BM with vFLIP. The lysates were also subjected to immunoblotting to examine expression of vFLIP and Flag for wild-type and deletion mutants of CADM1. Error bars represent s.e.m. of triplicates.

Previous studies have shown that NF-κB is chronically activated in KSHV-infected cells [15]. Therefore, we initially examined whether CADM1 is required for KSHV-mediated NF-κB activation. The endogenous CADM1 expression was stably suppressed with lentiviral shRNA followed by KSHV infection in HeLa cells, which were then transfected with NF-κB luciferase plasmids containing NF-κB response elements. Surprisingly, KSHV-mediated activation of NF-κB was impaired in CADM1 knockdown HeLa cells as determined by NF-κB activation luciferase assays (S4A Fig). KSHV K13 encodes vFLIP, expressed during latency, which is responsible for the chronic activation of NF-κB [14]. Next, we determined the effect of different amounts of CADM1 expression on vFLIP-mediated NF-κB activation. HeLa cells were transfected with increasing amounts of either CADM1 or vFLIP plasmids together with an NF-κB luciferase reporter. As expected, we found that increasing the dose of CADM1 plasmid with a constant amount of vFLIP expression significantly enhanced NF-κB activation compared to vFLIP alone. The effects appeared to be additive. Increasing the dose of vFLIP plasmid with a constant amount of CADM1 expression led to a plateau of NF-κB activation (Fig 2A). These results suggest that vFLIP-mediated NF-κB activation is dependent on CADM1 expression levels. We then determined the functional effect of endogenous CADM1 expression on vFLIP-mediated NF-κB activation by stably suppressing CADM1 expression with three different lentiviral shRNAs in HeLa cells as described previously [44], followed by transfection with vFLIP and NF-κB luciferase plasmids. As expected, the suppression of CADM1 expression by shRNA#3 significantly impaired vFLIP-mediated NF-κB activation in HeLa cells as determined by NF-κB luciferase assays (Fig 2B). Suppression of CADM1 expression had no effect on vFLIP expression as confirmed by immunoblotting (Fig 2B). To further examine the requirement of CADM1 for vFLIP-mediated NF-κB activation, we next transfected *Cadm1*^*+/+*^ and *Cadm1*^*−/−*^ MEFs with vFLIP and NF-κB luciferase plasmids. vFLIP-mediated NF-κB activation was impaired in *Cadm1*^*−/−*^ MEFs as examined by luciferase assays (Fig 2C). To identify the motifs of CADM1 required for vFLIP to activate NF-κB, *Cadm1*^*−/−*^ MEFs were transfected with vFLIP together with either Flag-tagged wild-type or deletion mutants of CADM1 (Fig 2D) and we found that the cytoplasmic tail and PDZ-BM of CADM1 were required for vFLIP to activate canonical NF-κB (Fig 2E).

### CADM1 is required for KSHV vGPCR to activate NF-κB and NFAT

**Fig 3:**
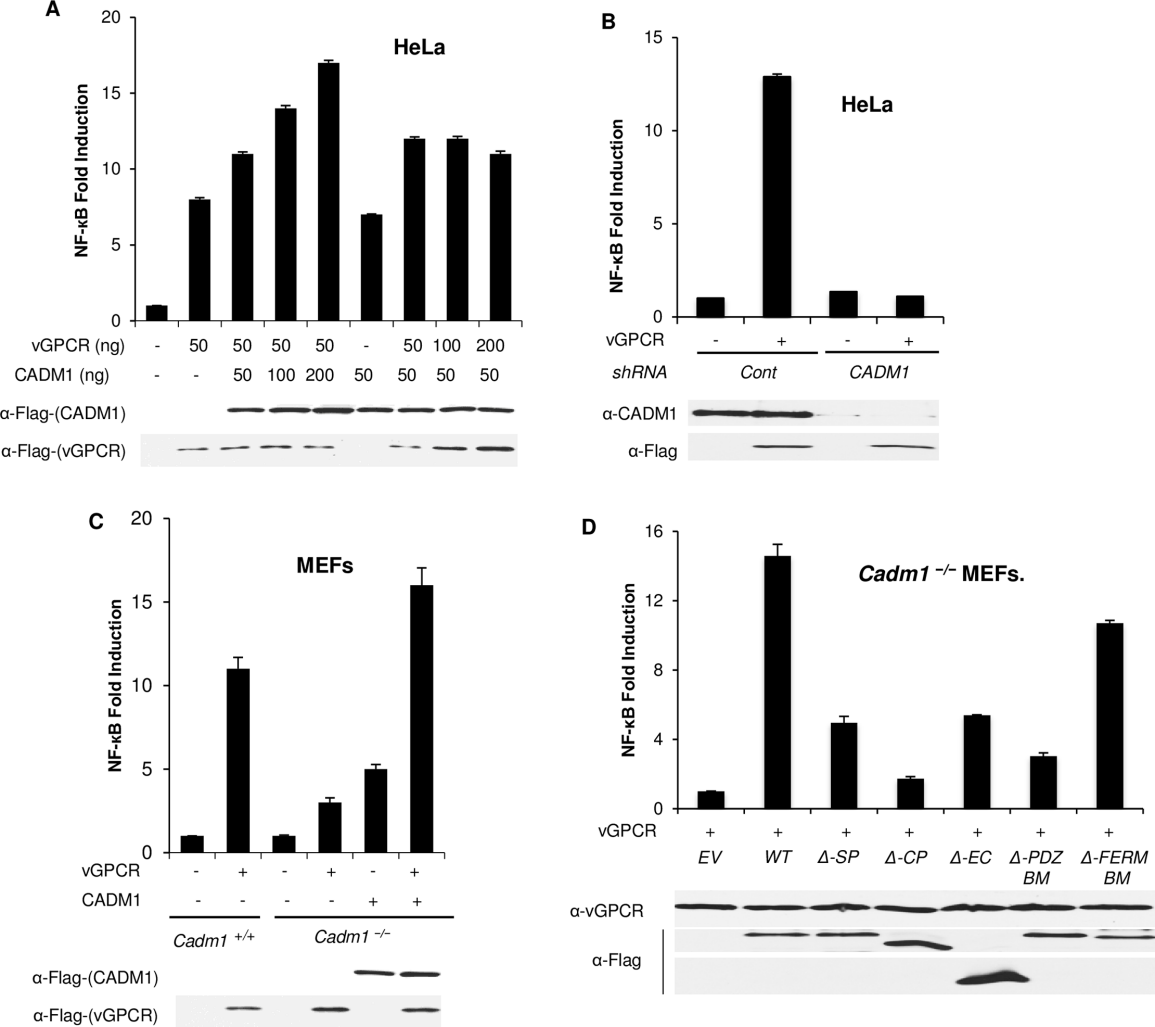
CADM1 is required for KSHV vGPCR to activate NF-κB. (A) Dual-luciferase assay using lysates of Hela cells expressing increasing amounts of CADM1 and vGPCR. HeLa cells were transfected with increasing amounts of either CADM1 or vGPCR with κB‐TATA Luc and pRL‐tk. After 36 hours, lysates were subjected to dual luciferase assays. The lysates were also subjected to immunoblotting to examine CADM1 and vGPCR expression using anti-Flag antibody. (B) HeLa cells expressing control scrambled shRNA or CADM1 shRNA were transfected with pRL-tk internal control Renilla luciferase plasmid, κB-TATA Luc and vGPCR as indicated. After 48 hours, lysates were subjected to dual luciferase assays. The lysates were also subjected to immunoblotting to examine vGPCR and CADM1 expression. (C) *Cadm1* ^*+/+*^, *Cadm1* ^*−/−*^, and *Cadm1* ^*−/−*^ MEFs reconstituted with wild-type Flag-CADM1 were transfected with vGPCR and κB‐TATA Luc and pRL‐tk plasmids. After 36 hours, lysates were subjected to dual luciferase assays. The lysates were also subjected to immunoblotting to examine CADM1 and vGPCR expression using anti-Flag antibody. (D) NF-κB luciferase assay of lysates of *Cadm1*^*−/−*^ MEFs transfected with an NF-κB firefly luciferase reporter and a renilla luciferase vector reporter together with empty vector or an expression vector for Flag-tagged wild-type CADM1, CADM1 ΔSP, CADM1 ΔCP, CADM1 ΔEC, CADM1 ΔPDZ-BM and CADM1 ΔFERM-BM with vGPCR. The lysates were also subjected to immunoblotting to examine expression of vGPCR and Flag for wild-type and deletion mutants of CADM1. Error bars represent s.e.m. of triplicates.

Previous studies showed that vGPCR is a membrane-associated protein that maintains chronic activation of NF-κB in PEL cell lines, primary B cells derived from KS patients, and endothelial cells [16, 29, 32, 57–59]. It has also been demonstrated that membrane-associated CADM1 protein interacts with several receptors and regulates their activities [60–62]. Thus, we next examined whether CADM1 played any role in vGPCR-induced NF-κB activation. To this end, we first transfected HeLa cells with either CADM1 or vGPCR plasmids in a dose-dependent manner. Increasing amounts of CADM1 expression with constant vGPCR expression significantly enhanced NF-κB activation compared to vGPCR alone, as measured by an NF-κB luciferase assay (Fig 3A). In contrast, constant CADM1 expression with increasing amounts of vGPCR expression had less of an effect on NF-κB activation compared to the highest dose of CADM1 with vGPCR expression (Fig 3A). Next, to examine whether CADM1 is required for vGPCR-mediated NF-κB activation, endogenous CADM1 expression was stably suppressed with lentiviral shRNA in HeLa cells as described previously [44], followed by transfection with vGPCR and NF-κB luciferase plasmids. As expected, vGPCR-mediated activation of NF-κB was impaired in HeLa cells after knockdown of CADM1 as determined by luciferase assays (Fig 3B). Next, we transfected *Cadm1*^*+/+*^, *Cadm1*^*−/−*^ and *Cadm1*^*−/−*^ MEFs reconstituted with wild-type Flag-CADM1 with vGPCR and NF-κB luciferase plasmids. vGPCR-mediated NF-κB activation was impaired in *Cadm1*^*−/−*^ MEFs compared to wild-type MEFs. Loss of vGPCR-mediated NF-κB activation in *Cadm1*^*−/−*^ MEFs was rescued by reconstituting wild-type CADM1 (Fig 3C). Next, to identify the motifs of CADM1 required for vGPCR to activate NF-κB, *Cadm1*^*−/−*^ MEFs were transfected with vGPCR, and reconstituted with either wild-type or deletion mutants of CADM1. We found that the cytoplasmic tail and PDZ-BM of CADM1 were required for vGPCR to activate canonical NF-κB (Fig 3D). Previous studies have demonstrated that vGPCR-mediated activation of Rac1 is critical for the activation of NF-κB [35, 63]. Therefore, we transfected *Cadm1*^*+/+*^ and *Cadm1*^*−/−*^ MEFs with a vGPCR plasmid to determine whether CADM1 has a role in vGPCR-induced Rac1 activation. Cell lysates from *Cadm1*^*+/+*^ and *Cadm1*^*−/−*^ MEFs expressing vGPCR were used to perform Rac1 pull-down assays. Surprisingly, the activation of Rac1 by vGPCR was significantly reduced in *Cadm1*^*−/−*^ MEFs compared to wild-type MEFs (S5 Fig).

Previous studies have also demonstrated that chronic activation of the transcription factor NFAT by vGPCR is critical for tumor formation [36, 37]. Therefore, we next measured NFAT-driven luciferase activity in the lysates of vGPCR transfected *Cadm1*^*+/+*^, *Cadm1*^*−/−*^, and *Cadm1*^*−/−*^ MEFs reconstituted with CADM1. Surprisingly, the vGPCR-mediated NFAT activation was decreased significantly in *Cadm1*^*−/−*^ MEFs compared to wild-type MEFs (S6 Fig). Reconstitution of *Cadm1*^*−/−*^ MEFs with wild-type CADM1 restored vGPCR-mediated NFAT activation (S6 Fig). These results suggest that CADM1 is critical for both vGPCR-mediated NF-κB and NFAT activation.

### CADM1 is essential for chronic activation of NF-κB in KSHV-associated PEL cell lines

**Fig 4:**
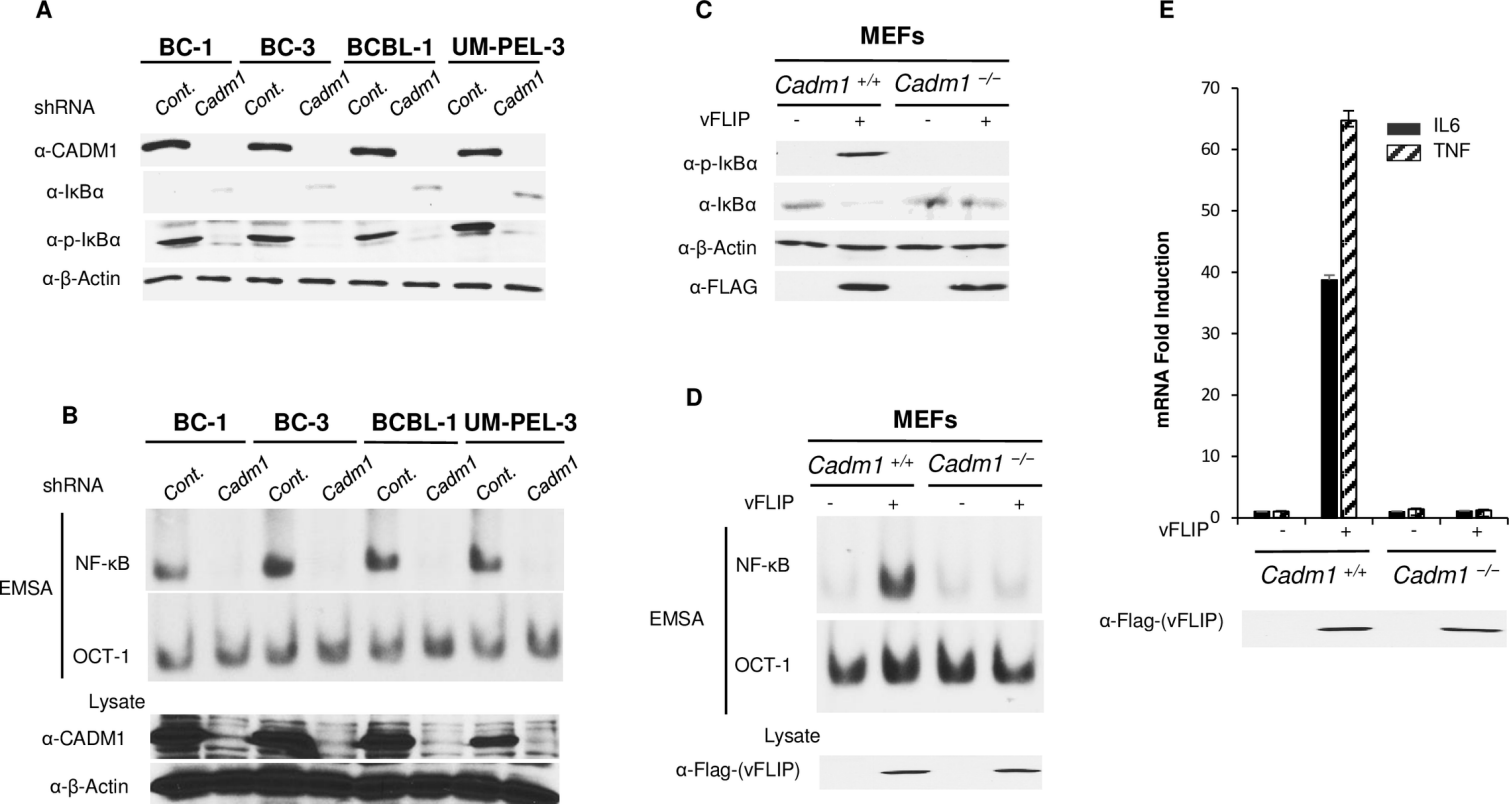
CADM1 expression is required for vFLIP-induced NF-κB activation. (A) Lysates from PEL cell lines (BC-1, BC-3, and BCBL-1) stably expressing control scrambled shRNA or CADM1 shRNA were subjected to immunoblotting with total anti-IκBα, anti-phospho-IκBα, anti-CADM1, and anti-β-actin antibodies. (B) Nuclear extracts from PEL cell lines (BC-1, BC-3, and BCBL-1) stably expressing control scrambled shRNA or CADM1 shRNA were used for NF-κB and Oct-1 EMSA, and cytoplasmic extracts were subjected to immunoblotting with anti-CADM1 and anti-β-actin antibodies. (C) Primary *Cadm1*^*+/+*^ and *Cadm1*^*−/−*^ MEFs were transfected with vFLIP plasmid. After 48 h, lysates were subjected to immunoblotting with anti-phospho-IκBα, anti-CADM1, and anti-Flag antibodies. (D) Nuclear extracts from primary *Cadm1*^*+/*+^ and *Cadm1*^*−/−*^ MEFs transfected with vFLIP were used for NF-κB and Oct-1 EMSA, and cytoplasmic extracts were subjected to immunoblotting with anti-Flag antibody. (E) Quantitative real-time PCR (qRT-PCR) analysis of *Tnf and Il-6* from *Cadm1*^*+/*+^ and *Cadm1*^*−/−*^ MEFs expressing vFLIP for 48 h. Lysates were subjected to immunoblotting with anti-Flag for vFLIP protein expression.

Recent studies have reported that the expression of vFLIP and vGPCR in both PEL cell lines (BC-1, BC-3, and BCBL-1) and primary B cells derived from KS patients is critical for chronic NF-κB activation and survival of PEL cells [14, 16, 64, 65]. Therefore, we examined NF-κB activation by analyzing the phosphorylation of IκBα and NF-κB DNA binding in KSHV-associated PEL cell lines after shRNA-mediated knockdown of CADM1, as well as NF-κB target gene induction in either vFLIP or vGPCR-expressing *Cadm1*^*−/−*^ MEFs and wild-type MEFs. The results clearly indicated that knockdown of CADM1 led to loss of IκBα phosphorylation and NF-κB DNA binding in PEL cell lines (Fig 4A and 4B), and vFLIP and vGPCR-expressing *Cadm1*^*−/−*^ MEFs compared to wild-type MEFs (Fig 4C and 4D and S7A and S7B Fig). A control Oct-1 electrophoretic mobility shift assay (EMSA) demonstrated similar DNA binding in all of the nuclear extracts. We next used qRT-PCR to examine the effect of CADM1 on vFLIP and vGPCR-induced NF-κB target genes such as *Tnf and Il-6*. The expression of *Tnf and Il-6* induced by vFLIP and vGPCR were impaired in *Cadm1*^*−/−*^ MEFs compared to wild-type MEFs (Fig 4E and S7C Fig). Next, we determined whether TNFα-induced NF-κB activation was also affected in *Cadm1*^*−/−*^ MEFs. *Cadm1*^*+/+*^ and *Cadm1*^*-/-*^ MEFs were transfected with either empty vector or CADM1 and then stimulated with TNFα for luciferase assays. NF‐κB activation by TNFα in *Cadm1*^*−/−*^ MEFs was not significantly affected compared to wild-type MEFs (S8 Fig). To determine whether CADM1 activates NF-κB upstream or downstream of the IKK complex, we transfected *Cadm1*^*+/+*^ and *Cadm1*^*-/-*^ MEFs with plasmids encoding either vGPCR, vFLIP, a constitutively active IKK beta (IKK-EE), CARD11, or p65 and performed qRT-PCR for NF-κB-target genes, *Tnf and Il-6*. As expected, vGPCR and vFLIP-induced *Tnf and Il-6* induction were impaired in *Cadm1*^*-/-*^ MEFs compared to wild-type MEFs (S9 Fig). Also, IKK-EE and p65-induced *Tnf and Il-6* mRNA expression were not significantly different in *Cadm1*^*+/+*^ and *Cadm1*^*-/-*^ MEFs (S9 Fig). Remarkably, CARD11-induced *Tnf and Il-6* mRNA expression was significantly impaired in *Cadm1*^*-/-*^ MEFs compared to wild-type MEFs (S9 Fig). These results suggest that CADM1 is also critical for CARD11-mediated *Tnf and Il-6* induction.

Previous studies have demonstrated that vFLIP is involved in constitutively inducing p100 processing to p52 [22, 24]. Therefore, we next determined whether CADM1 was required for vFLIP to induce p100 processing to p52. When BC-1, BC-3, and BCBL-1 were stably knocked down for CADM1, we found that vFLIP-mediated processing of p100 to p52 was completely impaired in CADM1 knockdown cells compared to cells expressing control shRNA (S10A Fig). The p100 processing to p52 by vFLIP was also impaired in *Cadm1*^*−/−*^ MEFs compared to wild-type MEFs (S10B Fig). These results strongly suggest that CADM1 is essential for constitutive activation of both NF-κB pathways in PEL cells.

### CADM1 interacts with KSHV oncogenes vFLIP and vGPCR

**Fig 5:**
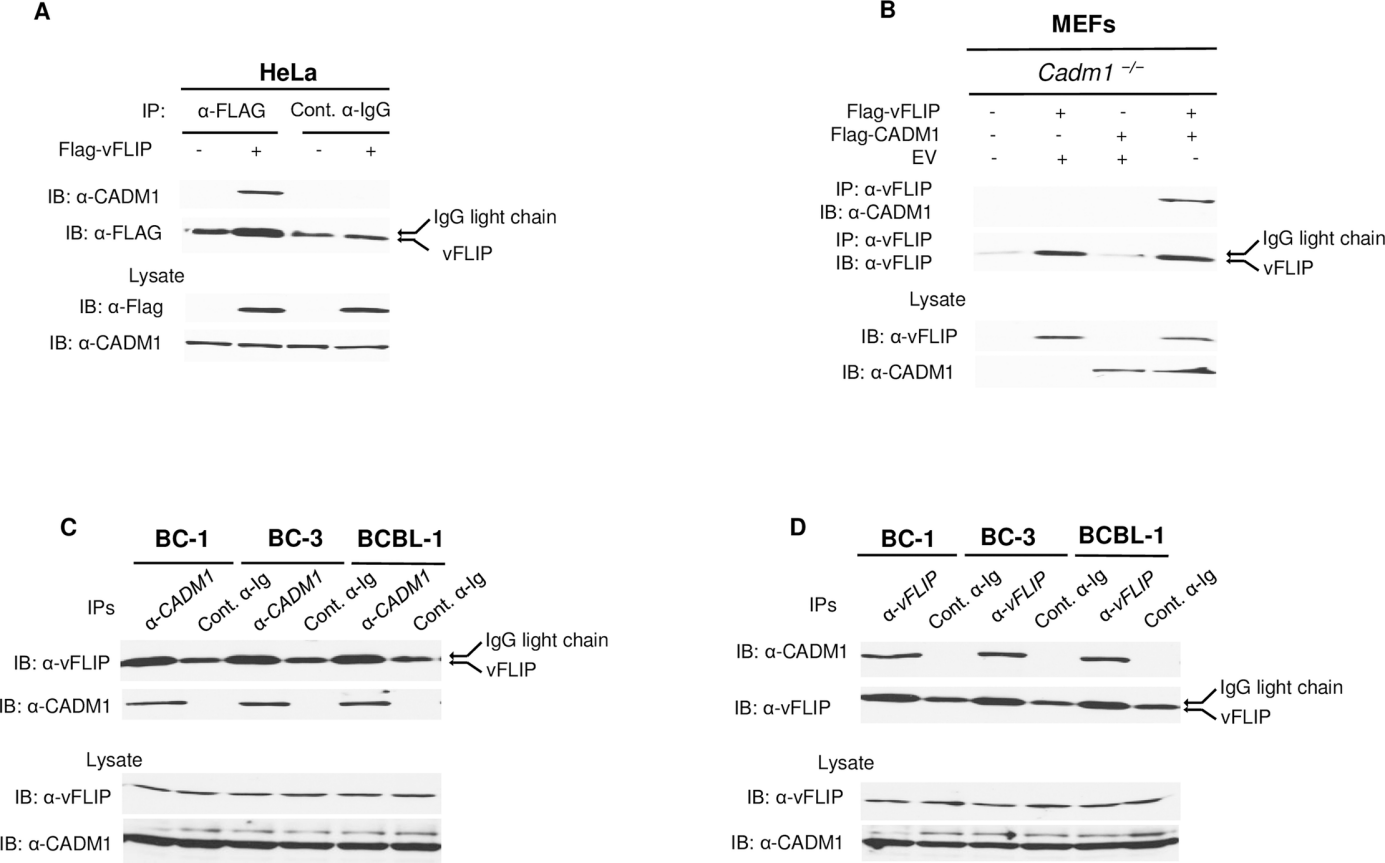
vFLIP interacts with CADM1. (A) HeLa cells were transfected with Flag-vFLIP. After 48 hours, cells were lysed and immunoprecipitated with either anti-Flag or control anti-IgG, followed by immunoblotting with anti-CADM1 and anti-Flag antibodies (Flag-vFLIP co-migrated with the IgG light chain). Lysates were examined for Flag-vFLIP and CADM1 expression. (B) Primary *Cadm1*^*−/−*^ MEFs were transfected with Flag-vFLIP expression vector, with or without Flag-CADM1. After 48 hours post-transfection, lysates were immunoprecipitated with anti-vFLIP and detected by immunoblotting with anti-CADM1 and vFLIP antibodies. Lysates were immunoblotted with anti-vFLIP and anti-CADM1 antibodies. (C) Lysates from PEL cell lines (BC-1, BC-3, and BCBL-1) were immunoprecipitated with either anti-CADM1 or control anti-IgG, followed by immunoblotting with anti-vFLIP and anti-CADM1. Lysates were examined for vFLIP and CADM1 expression. (D) Lysates from PEL cell lines (BC-1, BC-3, and BCBL-1) were immunoprecipitated with either anti-vFLIP or control anti-IgG, followed by immunoblotting with anti-CADM1 and anti-vFLIP. Lysates were examined for vFLIP and CADM1 expression.

Since CADM1 was required for vFLIP and vGPCR to activate NF-κB, we next performed co-immunoprecipitation (Co-IP) assays to determine if endogenous CADM1 interacts with vFLIP and vGPCR. HeLa cells were transfected with either vFLIP or vGPCR for co-IP assays to examine a potential interaction with endogenous CADM1. Indeed, endogenous CADM1 interacted with vFLIP and vGPCR (Fig 5A and S11A Fig), and the interaction was specific since no binding was observed when immunoprecipitations were performed with a control mouse immunoglobulin antibody (Fig 5A and S11A Fig). We next transfected *Cadm1*^*−/−*^ MEFs with CADM1 and vFLIP or vGPCR plasmids, and lysates were subjected to co-IP with anti-vFLIP or vGPCR antibodies to examine an interaction with CADM1. As expected, CADM1 interacted with both vFLIP and vGPCR in reconstituted MEFs (Fig 5B and S11B Fig). Since NF-κB is chronically activated in vFLIP and vGPCR-expressing PEL cells, and is impaired upon knockdown of CADM1, we examined CADM1 interaction with vFLIP and vGPCR in the PEL cell lines. Indeed, vFLIP and vGPCR are physically associated with CADM1 in BC-1, BC-3, and BCBL-1, cell lines (Fig 5C and S11C Fig). We also performed the reciprocal immunoprecipitation whereby vFLIP antibody was used for the immunoprecipitation, followed by immunoblotting with anti-CADM1 and obtained similar results (Fig 5D). Since the cytoplasmic tail (CP) and PDZ binding domain of CADM1 are critical for vGPCR and vFLIP-mediated NF-κB activation, we next determined if CP and PDZ domains of CADM1 are also required for the interaction with vGPCR and vFLIP. As expected, CADM1 was not able to interact with either vGPCR or vFLIP when CP or PDZ domains were deleted (S11D and S12 Figs). These results suggest that the PDZ binding domain of CADM1 is essential for the interactions with vFLIP and vGPCR in KSHV-infected cells.

### CADM1 is required for vFLIP and NEMO interaction and IKK complex activation in the plasma membrane lipid rafts

**Fig 6:**
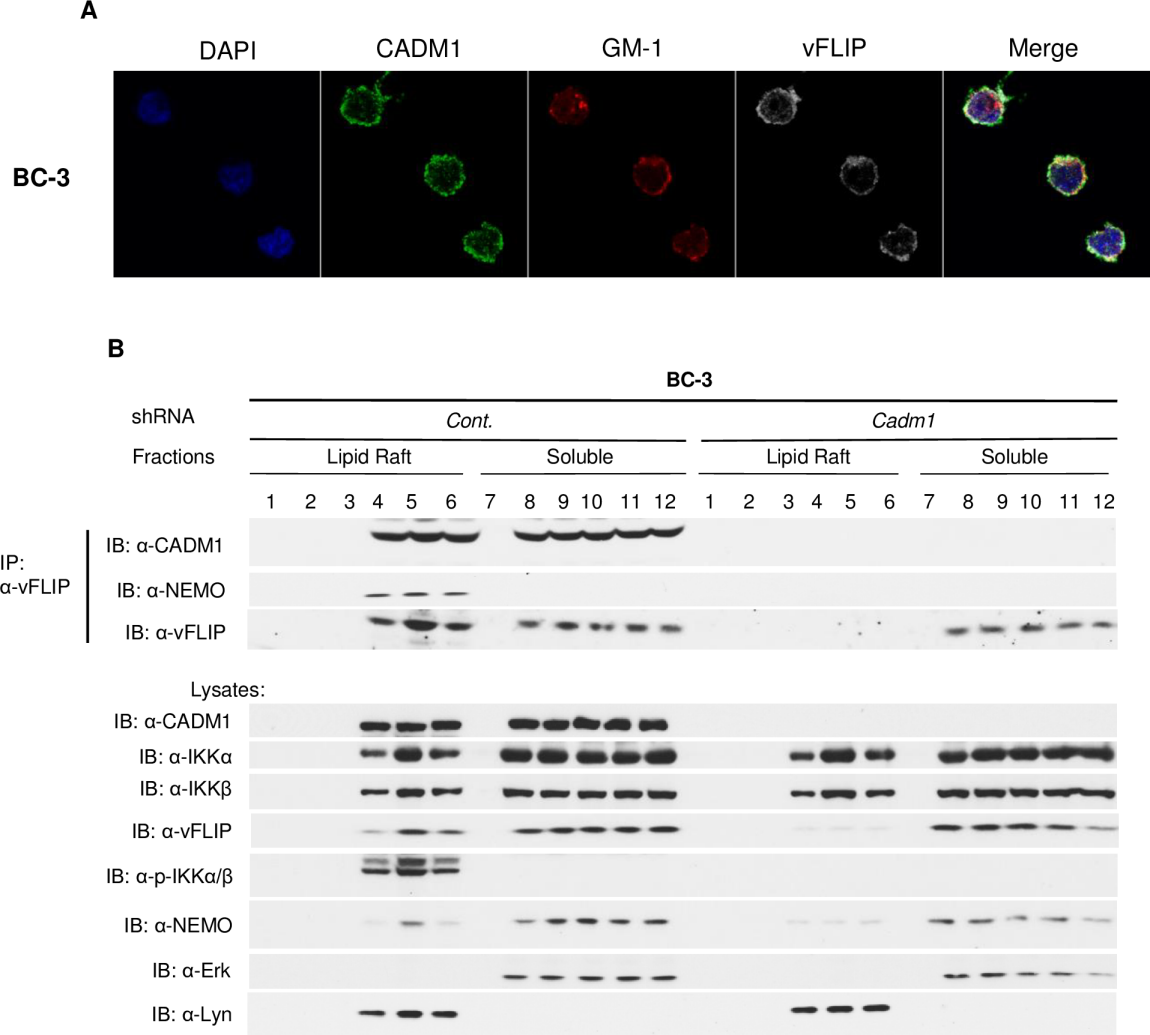
Membrane-associated CADM1 mediates vFLIP and NEMO interactions and IKK complex activation in lipid rafts. (A) BC-3 cells were stained with DAPI, anti-vFLIP, anti-CADM1, and cholera toxin B conjugated with red fluorescence to detect GM-1 and subjected to confocal microscopy. (B) Lipid raft fractionations from BC-3 cells stably expressing control scrambled shRNA or CADM1 shRNA were subjected to immunoprecipitation with anti-vFLIP. Samples immunoprecipitated with anti-vFLIP were immunoblotted with anti-vFLIP, anti-NEMO, and anti-CADM1. Lysates from lipid rafts fractions were examined for vFLIP, phospho-IKKα/β, total IKKα, IKKβ, NEMO, CADM1, ERK1 (marker for soluble fractions), and Lyn (lipid raft protein marker).

Previous studies have showed that CADM1 was localized in membrane lipid rafts as well as in the cytoplasm [44, 53, 66]. To further characterize the CADM1 and vFLIP cellular localization, we used confocal immunofluorescence imaging. As expected, vFLIP and CADM1 colocalized, and the proteins were found in lipid rafts as visualized by cholera toxin B staining of GM1 gangliosides in PEL (BC-3 and BCBL-1) cell lines (Fig 6A and S13A Fig). Thus, significant amounts of vFLIP co-localizes with CADM1 in the lipid rafts. Previous studies have demonstrated that IKK complex activation is initiated in membrane lipid rafts after receptor stimulation [67–69]. The viral oncogene Tax of HTLV-1 and LMP-1 of EBV are also localized in membrane lipid rafts where they activate the IKK complex and NF-κB. HTLV-1 Tax interacts with IKKγ (NEMO) in the membrane lipid rafts to promote IKK and NF-κB activation. Interestingly, vFLIP association with IKKγ (NEMO) is critical for NF-κB activation [70, 71]. Therefore, we prepared lysates from BC-3 PEL cells stably expressing lentiviral control shRNA or CADM1 shRNA and subjected these lysates to density gradient ultracentrifugation. Lysates were then used for immunoprecipitation with anti-vFLIP antibody to determine if the vFLIP-NEMO interaction required CADM1, and also if the vFLIP-NEMO interaction occurred in membrane lipid rafts or cytoplasm. As shown in Fig 6B, vFLIP interacted with NEMO only in lipid rafts corresponding to the fractions with the lipid raft markers Lyn (fractions 4, 5 and 6) from BC-3 PEL cells expressing control shRNA; however, vFLIP was unable to interact with NEMO in CADM1 knockdown cells. Immunoblot analysis of NEMO, CADM1 and total and phosphorylated IKKα/β from lysate fractions from BC-3 PEL cells revealed that IKKα/β was robustly phosphorylated in cells expressing control shRNA lipid raft fractions (fractions 4, 5 and 6); however, IKK activation was impaired in the lipid raft fractions (fractions 4, 5 and 6) from BC-3 PEL cells expressing CADM1 shRNA (Fig 6B).

Next, we determined whether vFLIP, NEMO, and CADM1 interactions in BC3 cells occurred in the presence of the cholesterol-chelating agent, methyl-β-cyclodextrin (MβCD) − a selective cholesterol inhibitor that impairs formation of lipid rafts. As expected, disruption of membrane lipid rafts with MβCD treatment significantly reduced the lipid raft ring formation as visualized by cholera toxin B staining of GM1 gangliosides in PEL (BC-3 and BCBL-1) cell lines (S13B and S14A Figs). Furthermore, lysates obtained from density gradient ultracentrifugation were immunoprecipitated as described above. As expected IKK activation, and vFLIP interaction with NEMO and CADM1 were intact in the membrane lipid rafts of control treated (media) BC-3 PEL cells. However, IKK activation, and vFLIP interaction with NEMO and CADM1 were impaired in the MβCD treated BC-3 PEL cells (S14B Fig).

### CADM1 is essential for the survival of KSHV-associated PEL cells

**Fig 7:**
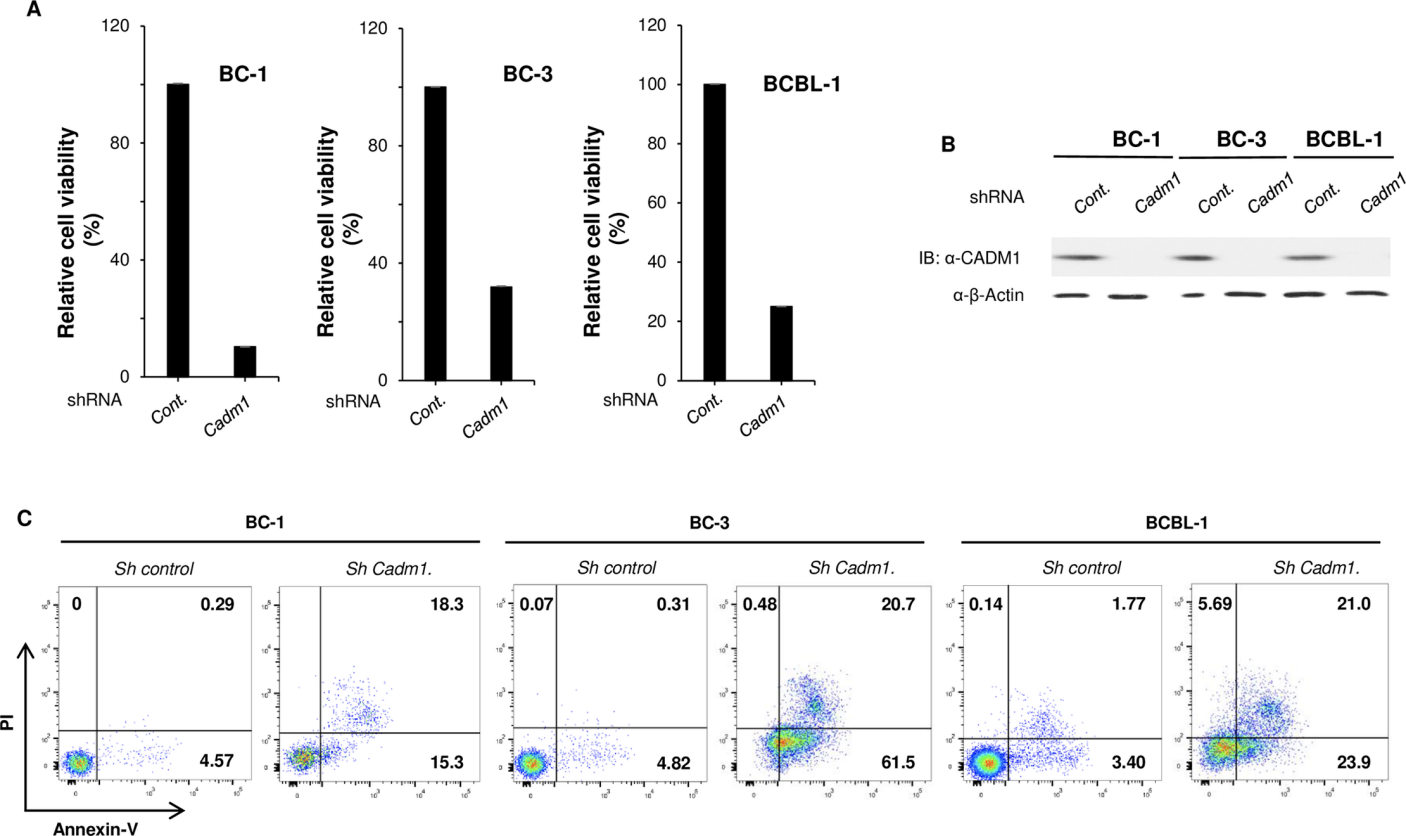
CADM1 is required for PEL cell survival. (A) Requirement of CADM1 for the viability of PEL cell lines. Cell viability assay was performed 96 hours after BC-1, BC-3, and BCBL-1 cells were transduced with lentiviruses expressing the indicated shRNAs. Relative cell viability (%) was expressed as a percentage relative to the control cells. (B) CADM1 protein was knocked down in BC-1, BC-3, and BCBL-1 cells after lentiviral transduction expressing the indicated shRNAs. Immunoblotting was performed with whole cell lysates. (C) Flow cytometric analysis of PEL cell lines transduced with shRNAs as described in (A). Cells were stained with both annexin-V-Alexa Fluor 488 and propidium iodide (PI). The distribution of cells is indicated as a percentage in each quadrant.

**Fig 8:**
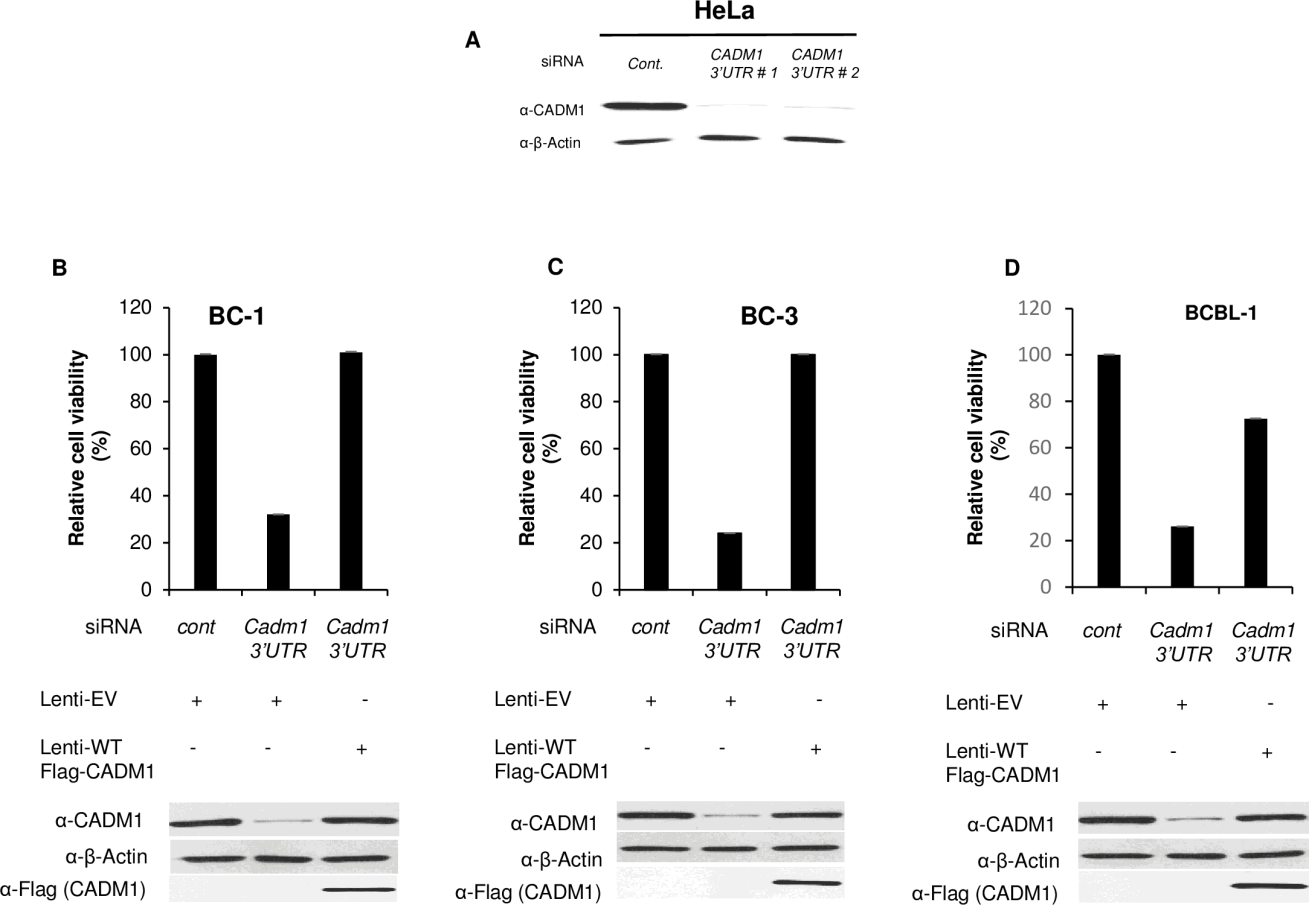
Lentiviral‐mediated transfer of wild-type CADM1 in CADM1-depleted PEL cells restores cell viability. (A) Suppression of CADM1 expression after 3’UTR CADM1 siRNA transfection in HeLa cells. Lysates were immunoblotted with anti-CADM1 and anti-β‐actin antibodies. (B-D) BC-1, BC-3, and BCBL-1 cells were transfected with 3’UTR CADM1 siRNA followed by transduction of lentiviruses containing wild-type Flag-CADM1 as indicated. Relative cell viability (%) was expressed as a percentage relative to the control cells. Whole cell lysates were immunoblotted with anti-CADM1, anti-β‐actin, and anti-Flag antibodies.

Ample evidence suggests that NF-κB-mediated induction of anti-apoptotic genes plays a critical role in the survival of tumor cells [72–74]. Given the essential role of CADM1 in chronic activation of NF-κB in PEL cells, we hypothesized that CADM1 may play a critical pro-survival role in PEL cells. Therefore, we used lentiviral shRNA to knock down CADM1 in BC-3 cells and determined the time course of cell viability and NF-κB activation. Interestingly, IκBα phosphorylation and degradation and cell viability began to decline significantly after 48 hours of knockdown of CADM1 expression in BC-3 cells as shown by immunoblotting and CellTiter-Glo Luminescent Cell Viability Assays (S15 Fig). However, there was no decline in IκBα phosphorylation and degradation and cell viability in control shRNA expressing BC-3 cells (S15 Fig). Next, we investigated BC-1, BC-3, and BCBL-1 PEL cell viability and cell death after lentiviral shRNA-mediated knockdown of CADM1. As expected, shRNA-mediated knockdown of CADM1 resulted in a significant loss of viability of BC-1, BC-3, and BCBL-1 PEL cells as shown by CellTiter-Glo Luminescent Cell Viability Assays after 96 hours (Fig 7A). shRNA-mediated CADM1 knockdown was validated by immunoblotting (Fig 7B). To determine if the loss of cell viability was due to apoptosis, CADM1 was knocked down in BC-1, BC-3, and BCBL-1 PEL cells and they were analyzed by Annexin V and propidium iodide (PI) staining; CADM1 knockdown resulted in significant apoptotic cell death in PEL cells after 96 hours (Fig 7C). To determine if CADM1 knockdown affects the cell viability and survival of non-infected BJAB cells, shRNA-mediated CADM1 knockdown was performed in BJAB cells and cell viability and cell death were analyzed as described above (S16A–S16C Fig). There was no effect of shRNA-mediated CADM1 knockdown on BJAB cells after 96 hours (S16A–S16C Fig). These results suggest that the suppression of CADM1 expression specifically affected KSHV-infected cells. We next determined the specificity of CADM1 knockdown on the cell death observed in BC-1, BC-3, and BCBL-1 PEL cells. BC-1, BC-3, and BCBL-1 cells were transfected with an siRNA specific for the 3’-untranslated region (UTR) sequence of CADM1 to allow for rescue experiments with ectopically expressed CADM1. First, we transfected two different CADM1 3’UTR siRNAs in HeLa cells to determine the efficiency of CADM1 knockdown. Both CADM1 3’UTR siRNAs efficiently knocked down CADM1 expression in HeLa cells (Fig 8A). To determine the specificity of CADM1 silencing on PEL (BC-1, BC-3, and BCBL-1) cell line viability, these cell lines were infected with lentiviruses containing either empty vector (EV) or wild-type Flag-tagged CADM1 followed by transfection with CADM1 3’UTR siRNA #2. As expected, knockdown of CADM1 by 3’UTR siRNA #2 significantly reduced cell viability as shown by CellTiter-Glo Luminescent Cell Viability Assays after 96 hours (Fig 8B–8D). Reconstitution of CADM1 expression by lentivirus containing wild-type CADM1 significantly restored BC-1, BC-3, and BCBL-1 cell viability (Fig 8B–8D). Furthermore, we analyzed cell death via Annexin V and propidium iodide (PI) staining using the BC-1 cells from above. Suppression of CADM1 expression by CADM1 3’UTR siRNA significantly increased apoptotic cell death compared to control siRNA expressing cells (S17 Fig). Restoration of CADM1 expression by lentiviruses containing wild-type CADM1 led to significantly reduced cell death compared to cells expressing CADM1 3’UTR siRNA (S17 Fig).

## Discussion

Although ample studies have demonstrated that CADM1 expression is silenced by promoter methylation in many solid tumors such as lung, melanoma, pancreatic, esophageal, uterine, and cervical cancer [75–79], its mechanistic functional roles as a tumor suppressor are unknown. Intriguingly, there are also a few exceptions whereby CADM1 is overexpressed in select tumor types, such as ATL and lung adenocarcinoma [49, 80, 81]. Our previous studies have described a possible mechanistic role of CADM1 in the chronic activation of NF-κB, which may provoke HTLV-I-associated ATL [44]. However, the expression and functional roles of CADM1 in other viral-induced leukemias and lymphomas are not yet clear. Persistent activation of NF-κB is critical for the survival of most types of leukemias and lymphomas, including PEL cells [16, 57, 58, 82]. Our current study has provided a link between CADM1 and NF-κB activation in KSHV-infected PEL cells.

Our findings in the current study demonstrate that the expression of CADM1 is induced by KSHV infection at a very low (0.1) MOI in primary human B-cells. Also, CADM1 expression was upregulated by several folds in PEL (BC-1, BC-3, BCBL-1, and UM-PEL-3) cell lines. These results are in line with previously published results whereby CADM1 expression was induced by the oncogenic virus HTLV-1 in primary human T-cells [49, 81]. Our previous findings suggested that CADM1 expression was critical for the HTLV-1 oncogene Tax to maintain chronic NF-κB activation. Furthermore, CADM1 facilitates Tax ubiquitination by augmenting Tax and ubiquitin-conjugation enzyme 13 (Ubc13) interaction in HTLV-1-infected cells[44]. Thus, CADM1 likely serves unique roles in NF-κB signaling and tumorigenesis by oncogenic viruses. The findings from our current study demonstrate that the PDZ binding motif of CADM1 located at the cytoplasmic tail is essential for both vFLIP and vGPCR to maintain chronic activation of NF-κB. The PDZ binding domain of CADM1 is essential to interact with vFLIP and vGPCR.

Previous studies have demonstrated that the interaction between NEMO-vFLIP or NEMO-Tax is crucial for maintaining chronic activation of NF-κB. HTLV-1 Tax interacts with NEMO and activates IKK in membrane lipid rafts [44, 45, 83]. However, it was unclear how vFLIP activates the IKK complex. Our results strongly suggest that CADM1, vFLIP and NEMO form a complex in plasma membrane lipid rafts to promote IKK activation (Fig 6B). Disruption of membrane lipid rafts using methyl-β-cyclodextrin (MβCD), a selective cholesterol inhibitor, impaired CADM1, vFLIP and NEMO interactions and NF-κB activation as well (S14B Fig). A recent study has demonstrated that vFLIP interacts with MALT-1 (mucosa-associated lymphoid tissue protein-1, also known as paracaspase) to activate NF-κB in PEL cells [84]. MALT-1 is also known to be associated with membrane lipid rafts to initiate IKK activation in TCR and BCR signaling pathways [85, 86]. Therefore, it will be important in future studies to determine whether CADM1 is essential for the MALT-1-vFLIP interaction in PEL cells. In agreement with this, our results indicate that membrane-associated CADM1 is essential for vFLIP to activate the IKK kinase complex in the membrane lipid rafts (S18 Fig). Normally, several cytoskeletal components and their binding partners play critical roles in assembling membrane lipid rafts in receptor-mediated signaling pathways. Therefore, it would be interesting to determine if CADM1 functions in association with cytoskeletal components to interact with vFLIP and NEMO and initiate IKK activation in the membrane lipid rafts [87].

Our results clearly indicate that CADM1 is required for vFLIP and vGPCR to maintain chronic NF-κB activation. Thus, it is likely that CADM1 may play crucial roles in KSHV-induced tumorigenesis and survival of PEL cells. It will be interesting to determine if CADM1 is also required for other KSHV oncogenes, such as K15, to maintain chronic activation of NF-κB and tumorigenesis. K15 is also expressed at the cell surface similar to vGPCR and activates NF-κB [58, 88]. Thus, it is possible that CADM1 may facilitate K15-mediated NF-κB activation. Although previous studies have demonstrated that vFLIP-mediated chronic activation of NF-κB occurs at the level of the IKK complex, where vFLIP directly interacts with NEMO, it is unclear how vFLIP maintains chronic NF-κB activation in the presence of strong vFLIP negative regulatory mechanisms [70, 89, 90].

Previous studies have demonstrated that the ubiquitin-editing enzyme A20/TNFAIP3 inhibits vFLIP-mediated NF-κB activation, and the ubiquitin ligase ITCH targets vFLIP for proteasomal degradation [89, 90]. Our previous studies have demonstrated that the A20 ubiquitin-editing enzyme complex (A20, ITCH and TAX1BP1) inhibits receptor-mediated NF-κB activation, which was prevented by CADM1 in HTLV-1 Tax-mediated NF-κB activation [44, 91, 92]. Thus, it will be interesting to determine in future studies if the abundantly expressed CADM1 would rescue vFLIP-mediated NF-κB activation in KSHV-infected cells. It is likely that other IKK kinase complex activating kinases such as TAK1, may also be involved in vFLIP and vGPCR-mediated NF-κB activation. Identification of other NF-κB activating molecules in vFLIP and vGPCR expressing cells will be of great interest for future studies.

The small GTPase, Rac1, has been shown to be critical for vGPCR to activate NF-κB [35]; however, the mechanism of IKK activation by Rac1 in vGPCR-expressing cells is poorly understood. Our finding suggests that CADM1 is essential for vGPCR to active Rac1. Thus, it is possible that CADM1 may recruit Rac1 to GPCR to activate the IKK complex (S18 Fig). Our results clearly suggest that CADM1 is required for vGPCR to activate NF-κB and NFAT. Therefore, it is most likely that CADM1 may be cooperating with Rac1 in vGPCR-expressing cells. In future studies, we will determine the mechanisms by which CADM1 facilitates Rac1 and vGPCR-mediated NF-κB and NFAT signaling pathways. Since our results reveal that the PDZ binding motif of CADM1 is critical for vGPCR-mediated NF-κB activation, it is possible that the PDZ binding motif of CADM1 may be involved in recruiting Rac1. Moreover, future studies will be focused on finding precise mechanisms required for vGPCR-mediated NF-κB and NFAT activation, of which ubiquitination may be important and represent potential drug targets for KSHV-associated malignancies. Although many studies have suggested that CADM1 functions as a tumor suppressor, our findings indicate that CADM1 functions as an oncogene/tumor promoter in KSHV-infected cells.

## Materials and methods

### Ethics statement

Blood from healthy individuals was purchased from Innovative Research, Novi, MI, USA., which is a commercial donor center licensed by the U.S.

### Biological reagents and antibodies

The PEL cell lines BC-1, BC-3, BCBL-1, and UM-PEL-3 were described previously [93]. PEL cell lines were cultured in RPMI medium (Mediatech, Inc.) supplemented with 10% fetal bovine serum, 100 U/ml penicillin, and 100 μg/ml streptomycin (Invitrogen, Carlsbad, CA). HeLa cells were cultured in complete DMEM medium (Mediatech, Inc.) containing 10% fetal bovine serum, heat inactivated, sterile-filtered (Sigma-Aldrich), L-glutamine, 1x penicillin-streptomycin (Invitrogen/Life Technologies). *Cadm1*^*+/+*^ and *Cadm1*^*−/−*^ MEFs were cultured in Dulbecco's modified Eagle's medium (Mediatech, Inc.) supplemented with 10% fetal bovine serum, 100 U/ml penicillin, and 100 μg/ml streptomycin. HUVECs were purchased from the ATCC and were incubated in endothelial growth medium (EGM-2) supplied with growth factors obtained from an EGM-2 Bullet kit (Lonza). The plasmids pCAGI-Puro-FLAG-CADM1, pCAGI-Puro-FLAG-CADM1-ΔCP (deletion of the cytoplasmic tail, aa 404–445), pCAGI-Puro-FLAG-CADM1-ΔEC (deletion of the extracellular region, aa 1–362), pCAGI-Puro-FLAG-CADM1-ΔFERM (deletion of the FERM domain-binding motif, aa 401–413), and pCAGI-Puro-FLAG-CADM1-ΔPDZ-BM (deletion of the PDZ domain-binding motif, aa 442–445) were described previously [61], and gift from Yoshimi Takai, Kobe, Japan. The p65 plasmid was gift from Dr. Edward Harhaj. NF-κB-TATA luciferase constructs have been described previously [92]. The constitutively active IKK beta (IKK-EE), CARD11 and NFAT Luc constructs were purchased from Addgene Inc., Cambridge, MA, USA. The Luciferase-based detection of NFAT was performed as described previously [94]. Cadm1 3’UTR siRNAs were purchased from Dharmacon, Lafayette, CO. The following antibodies were used in this study: anti-β-actin (Abcam), the monoclonal anti-vGPCR antibody was obtained from R & D System. The anti-ERK1/2, anti-phospho-IκBα, anti-phospho-IKKα/β (Cell Signaling Beverly, MA), anti-NEMO, anti-IKKα, anti-IKKβ, anti-IκBα (C-21), anti-IκBα, and anti-Lyn (Santa Cruz Biotechnology), Anti-Lana (KSHV) and anti-CADM1 antibodies were obtained from MBL International Corporation.

### Plasmid constructs, lentiviral particle production and target cell infection

vGPCR and vFLIP genes were PCR amplified from BC-3 cell cDNA, digested with *NotI* and *EcoRI*, and cloned into p3X‐Flag‐CMV‐7.1 (Sigma). To reconstitute CADM1 in PEL cells, Flag-CADM1 plasmid was used as a template for PCR-mediated cloning into the pDUET-GFP-hygromycin and pCDH-Cuo-MCS-EFI-GFP-T2A-puro lentiviral vector. All Flag-tagged clones were confirmed by DNA sequencing. A CADM1-specific shRNA construct was used to knockdown CADM1 expression as described previously [44]. Briefly, HEK 293-T-cells in 6-well culture plates were transfected with 1 μg of control scrambled shRNA or CADM1 shRNA with 2 μg of packaging plasmids (OriGene Technologies) containing a puromycin selection marker using FuGENE 6 (Roche). Seventy-two hours post-transfection, the supernatants were collected and concentrated by ultracentrifugation and the pellets were resuspended in ice-cold PBS. Viral stocks were used to infect HeLa and PEL (BC-1, BC-3, BCBL-1, and UM-PEL-3) cells and selected with puromycin. Lentiviruses expressing CADM1 or control (GFP) empty vector were generated as described above.

### Transfections and luciferase assays

Transient transfections in HeLa cells and PEL cells were performed with GenJet (SignaGen), FuGENE 6 and FuGENE HD (Roche, Indianapolis, IN) transfection reagents, respectively. For siRNA transfections, on day 1 cells were transfected with siRNA using Lipofectamine 2000 (Invitrogen, Carlsbad, CA). On day 2, other DNAs were transfected into the same cells with FuGENE 6 (Roche) or infected with lentiviruses expressing CADM1 or control (GFP) empty vector. Cells were harvested 24-96h post‐transfection/infection and cell lysates were prepared in either 1 × Passive Lysis Buffer (Promega, Madison, WI) or RIPA buffer. Luciferase activity was assayed using the Dual Luciferase Assay system according to the manufacturer's instructions (Promega, Madison, WI). Error bars indicate the standard error of the mean (s.e.m.) of triplicate samples from a representative experiment.

### Quantitative real‐time PCR

Total RNA was extracted using Trizol reagent (Invitrogen). Complementary DNA (cDNA) was synthesized using qScript cDNA SuperMix (Quanta Biosciences) and subjected to quantitative RNA polymerase chain reaction (PCR). The PCR reactions were performed on an ABI 7500 instrument and a 7300 Real Time PCR System (Applied Biosystems, Foster City, CA).

### Lipid raft isolation

Lipid raft fraction analysis was carried out as described previously [44]. Briefly, BC-3 cells were lysed in 2 ml of extraction buffer (20 mM Tris-Cl, pH 7.4, 150 mM NaCl, 1 mM EDTA, 1% Triton X-100 plus protease inhibitor cocktail). Lysates were combined with a 60% Optiprep solution to yield 40% and placed at the bottom of the ultracentrifuge tube followed by overlaying with an equal volume (4 ml) of discontinuous 30% and 5% OptiPrep Density Gradient medium. Samples were centrifuged at 100,000 × g for 4 hours at 4°C in an SW41 rotor. 1 ml of each fraction from the top to bottom was collected and equal volumes of each fraction were loaded onto SDS-PAGE gels. For the depletion of plasma and intracellular membrane cholesterol by MβCD, BC-3 cells cultured in RPMI medium supplemented with fetal bovine serum (10%) and penicillin-streptomycin (1%) were treated with or without 10 mM MβCD and incubated at 37°C for 30 min. Followed by this step lysates were subjected to density gradient ultracentrifugation for lipid raft fractionation analysis.

### Co-immunoprecipitation (Co-IP) assays

Ubiquitination assays were performed as described previously [91, 95]. Briefly, MEFs, HeLa or PEL (BC-1, BC-3, BCBL-1, and UM-PEL-3) cells were lysed in RIPA buffer and immunoprecipitated with either Flag, vFLIP, CADM1, or vGPCR antibodies. Immunoprecipitates were washed three times with respective buffers. Immunoblotting was performed with the indicated antibodies for co-IPs.

### Immunoblotting

Immunoblotting was done as described previously [96]. Whole‐cell lysates were resolved by SDS–PAGE, transferred to nitrocellulose membranes, blocked in 5% milk, incubated with appropriate primary and secondary antibodies, and detected by Western Lightning enhanced chemiluminescence reagent (Perkin Elmer, Boston, MA).

### Rac1 activity assay

The level of active Rac1 was assayed using a pull-down assay kit (cytoskeleton, Denver, CO, USA) following the manufacturer's instructions. Briefly, vGPCR expressing *Cadm1* ^*+/+*^ and *Cadm1* ^*−/−*^ MEFs were harvested and lysed in lysis buffer. Equal amounts of lysates were incubated with PAK-PBD beads at 4°C for 2 h. PAK-PBD beads were pelleted by centrifugation and washed with wash buffer. Active Rac1 was detected by western blotting.

### Purification of B cells

Primary human B-cells were isolated from whole blood using Ficoll-Paque PLUS (GE Healthcare) as previously described [97]. B cells were then isolated by negative selection using EasySep B-cell Isolation Kit (StemCell Technologies) according to the manufacturer’s instructions.

### Confocal microscopy

Confocal microscopy analysis was performed as described previously [44]. Briefly, the cells were seeded onto 12-mm poly-L-lysine-coated coverslips (BD Biosciences, Bedford, MA) and were briefly centrifuged prior to fixation. The cells were washed three times with PBS and fixed in 4% paraformaldehyde for 15 minutes at room temperature. The fixed cells were permeabilized with PBS containing 0.2% Triton X-100, and nonspecific binding was prevented by a 1 hour incubation in SuperBlock buffer (Thermo Scientific) followed by staining with primary antibodies: mouse anti-vFLIP, rabbit anti-CADM1 (Santa-Cruz Biotechnology), anti-chicken anti-CADM1 (MBL International Corporation), and GM1 (Abcam), diluted in PBS containing 1% BSA and incubated for 2 hours followed by five washes with PBS containing 1% BSA. Secondary antibodies were used as follows: Alexa Fluor 555- donkey anti-mouse IgG (for vFLIP), Alexa Fluor 488-donkey anti-rabbit IgG or donkey anti-chicken (for CADM1), Alexa Fluor 647-conjugated cholera toxin subunit B (Invitrogen), and Cy2 donkey anti-chicken IgG (for CADM1) (Jackson ImmunoResearch) were incubated for 45 min followed by four washes with PBS. The cells were then incubated with DAPI 500ng/ml (Sigma). After washing three times with PBS, the coverslips were mounted onto the glass slides with ProLong Gold anti-fade reagent (Invitrogen) and then observed with an SP5 confocal microscope (Leica).

### Electrophoretic mobility shift assay (EMSA)

Nuclear extracts were performed as described previously [98]. NF-κB activation was detected using LightShift Chemiluminescent EMSA Kit (Signosis) according to the manufacturer's instructions.

### Flow cytometric analysis

FACS analysis was performed as described previously [99]. Briefly, KSHV infected cell lines and PBMCs were treated with an antibody cocktail (α-CD3, α-B220 (BD Biosystems), α-CD19 (StemCell Technologies, and α-CADM1 (MBL International Corporation)) in 1X FACS buffer on ice for 20 min. Cells were then washed with 1X PBS and corresponding secondary antibodies were added to cells along with live/dead marker and incubated for 20 min on ice. Samples were washed with 1X PBS, fixed with 4% paraformaldehyde for 10 min on ice and washed with 1X FACS buffer, then resuspended in 1X FACS buffer. Data were recorded on the BD LSR II with FACSDiva software and were analyzed using FlowJo software.

### KSHV virus purification and infection

KSHV was isolated from BC-3 cells as previously described [100, 101] with slight modification. Briefly, TPA was added to BC-3 cell culture at 20 ng/mL for 48 hours to induce the KSHV lytic cycle. The culture media was centrifuged at 2,000 rpm for 15 min to remove cells and cell debris. The medium was filtered through 0.45μm pore-size filters. Then, the viral particles were concentrated by overnight mixing at 4^0^ C in 40% PEG (polyethylene glycol) media followed by centrifugation at 3500 rpm for 30–40 min. The pellet was resuspended in RPMI media and used to infect human primary B cells and HeLa cells.

### Cell apoptosis and viability assays

PEL (BC-1, BC-3, BCBL-1) cells were transduced with lentivirus expressing the indicated shRNAs for 96 hours, Annexin V/PI staining procedure was performed as described previously [102]. Briefly, cells were washed in phosphate buffered saline (PBS) and 1× Annexin V binding (AV) buffer (10 mM HEPES (pH 7.4), 140 mM NaCl, and 2.5 mM CaCl2) twice, and resuspended in 100 μl of AV buffer. The cells were incubated with Annexin V FITC (BD Bioscience) in the dark for 15 min. at room temperature and then PI (BD Bioscience) was added for an additional 15 min, fixed with 1% formaldehyde and treated with RNase A (50 μg/ml) for 15 min at 37°C. Data were recorded on the BD LSR II with FACSDiva software and were analyzed using FlowJo software. The CellTiter-Glo Luminescent Cell Viability Assay (Promega, Madison, WI) was used to determine cell viability.

### Generation of vFLIP monoclonal antibody

Antibody that recognizes the vFLIP protein was generated with a standard monoclonal antibody production protocol (GenScript USA Inc.). Monoclonal antibody production and purification from the supernatant of hybridoma cells were carried out by GenScript USA Inc., (NJ, USA), Monoclonal antibodies were analyzed by ELISA and Western Blot.

### Statistical analysis

Two-tailed unpaired T test was performed with Prism software. Error bars represent the standard deviation of triplicate samples. The level of significance was defined as: ***P<0.001, **P<0.01, *P<0.05.

## Supporting information

S1 Fig Induction of CADM1 mRNA in KSHV-infected HUVEC cells. HUVEC cells were infected (0.1 MOI) with KSHV. After 48 hours, total RNA was prepared and subjected to quantitative PCR for CADM1 mRNA. The lysates were also subjected to immunoblotting to examine CADM1, KSHV-associated protein, LANA and β-actin expression.
(TIF)

S2 Fig CADM1 protein expression in primary human B cells, PEL (BC-1, BC-3, and BCBL-1), and BJAB cells. CADM1 expression was assessed in primary human B cells, PEL cell lines, and non-infected BJAB cell lines by flow cytometry. Representative histograms are shown. Black dotted lines correspond to IgY control and Red color histograms correspond to CADM1 expression in primary human B cells (A), PEL cells BC-1 (B), BC-3 (C), BCBL-1(D), and BJAB (E). (F) MFI of CADM1 expression in primary human B cells, BC-1, BC-3, BCBL-1 and BJAB, respectively.
(TIF)

S3 Fig Induction of CADM1 mRNA by the KSHV oncogenes vGPCR and vFLIP. HeLa cells were transfected with vGPCR or vFLIP plasmids. After 48 hours, total RNA was prepared and subjected to quantitative PCR for CADM1 mRNA. The lysates were also subjected to immunoblotting to examine Flag-tagged, vGPCR and vFLIP expression.
(TIF)

S4 Fig Activation of NF-κB is impaired in the absence of CADM1 expression in HeLa cells infected with KSHV. NF-κB luciferase assay using lysates of HeLa cells expressing control scrambled shRNA or CADM1 shRNA (+/- infection with KSHV (0.1 MOI)) and transfected with pRL-tk internal control Renilla luciferase plasmid, κB-TATA Luc for 24 hours as indicated. After 24 hours of infection, lysates were subjected to dual luciferase assays. The lysates were also subjected to immunoblotting to examine CADM1, KSHV-associated protein, LANA, and β-actin expression.
(TIF)

S5 Fig CADM1 is required for vGPCR-induced Rac1 activation. Equal amount of lysates of *Cadm1* ^*+/+*^ and *Cadm1* ^*−/−*^ MEFs expressing vGPCR were incubated with PAK-PBD. Active Rac1, Flag-vGPCR expression, total Rac1, and β-actin were detected by western blotting.
(TIF)

S6 Fig CADM1 is required for vGPCR-mediated NFAT activation. *Cadm1*^*+/+*^, *Cadm1*^*-/-*^, and *Cadm1*^*-/-*^ MEFs reconstituted with wild-type Flag-tagged CADM1 were transfected with an NFAT-dependent luciferase reporter construct and vGPCR. After 36 hours, cells were lysed and subjected to immunoblotting to examine CADM1 and vGPCR expression using anti-Flag antibody.
(TIF)

S7 Fig CADM1 expression is required for NF-κB activation. (A) Primary *Cadm1*^*+/+*^ and *Cadm1*^*−/−*^ MEFs were transfected with vGPCR plasmid. After 48 h, lysates were subjected to immunoblotting with anti-phospho-IκBα, anti-CADM1, and anti-Flag antibodies. (B) Nuclear extracts from primary *Cadm1*^*+/*+^ and *Cadm1*^*−/−*^ MEFs transfected with vGPCR were used for NF-κB and Oct-1 EMSA, and cytoplasmic extracts were subjected to immunoblotting with anti-Flag antibody. (C) Quantitative real-time PCR (qRT-PCR) analysis of *Tnf and Il-6* from *Cadm1*^*+/*+^ and *Cadm1*^*−/−*^ MEFs expressing vGPCR for 48 hours. Lysates were subjected to immunoblotting with anti-Flag for vGPCR protein expression.
(TIF)

S8 Fig TNFα-mediated NF-κB activation is not impaired in *Cadm1* ^*−/−*^ MEFs. NF-κB luciferase assay using lysates of Cadm1^+/+^ and Cadm1^-/-^ MEFs transfected with either empty vector, CADM1, and κB‐TATA Luc and pRL‐tk and stimulated with TNFα for 8 hours. Lysates were subjected to dual luciferase assays. The lysates were also subjected to immunoblotting to examine CADM1, expression using anti-Flag antibody.
(TIF)

S9 Fig CADM1 functions upstream of the IKK complex. *Cadm1* ^*+/+*^ and *Cadm1* ^*−/−*^ MEFs were transfected with either Empty Vector, vFLIP, vGPCR, IKK(EE), CARD11, or p65. After 36 hours, total RNA was prepared and subjected to quantitative PCR for *Tnf* and *Il-6* mRNAs. The lysates were also subjected to immunoblotting to examine vFLIP, vGPCR, IKK, CARD11 and p65 expression using anti-Flag, anti-IKK, anti-Card11 and p65 antibodies, respectively.
(TIF)

S10 Fig vFLIP requires CADM1 to activate the non-canonical NF-κB pathway. (A) Cell lysates from BC-1, BC-3, and BCBL-1 cells transduced with lentiviruses expressing the indicated shRNAs, were subjected to immunoblotting with anti-p100/p52, anti-CADM1, and anti-β-actin antibodies. (B) Lysates from primary *Cadm1*^*+/+*^ and *Cadm1*^*−/−*^ MEFs transfected with vFLIP, immunoblotted with anti-Flag, anti-p100/p52, and anti-β-actin antibodies.
(TIF)

S11 Fig vGPCR interacts with CADM1. (A) HeLa cells were transfected with Flag-vGPCR. After 48 hours, cells were lysed and immunoprecipitated with either anti-Flag or control anti-IgG, followed by immunoblotting with anti-CADM1 and anti-Flag antibodies. Lysates were examined for Flag-vGPCR and CADM1 expression. (B) Primary *Cadm1*^*−/−*^ MEFs were transfected with Flag-vGPCR expression vector, with or without Flag-CADM1. After 48 hours post-transfection, lysates were immunoprecipitated with anti-vGPCR and detected by immunoblotting with anti-CADM1 and vGPCR antibodies. Lysates were immunoblotted with anti-vGPCR, and anti-CADM1 antibodies. (C) Lysates from PEL cell lines (BC-1, BC-3, BCBL-1, and UM-PEL-3) were immunoprecipitated with either anti-CADM1 or control anti-IgG, followed by immunoblotting with anti-vGPCR and anti-CADM1. Lysates were examined for vGPCR and CADM1 expression. (D) Mapping the interaction between CADM1 and vGPCR. HeLa cells were transfected with vGPCR with the indicated Flag-CADM1 mutants. After 36 hours post-transfection, lysates were immunoprecipitated with anti-vGPCR and detected by immunoblotting with anti-Flag and anti-vGPCR antibodies. Lysates were immunoblotted with anti-Flag antibody.
(TIF)

S12 Fig Mapping the interaction between CADM1 and vFLIP. HeLa cells were transfected with a vFLIP expression vector together with the indicated Flag-CADM1 mutants. After 36 hours post-transfection, lysates were immunoprecipitated with anti-vFLIP and detected by immunoblotting with anti-Flag and anti-vFLIP antibodies. Lysates were immunoblotted with anti-Flag antibody.
(TIF)

S13 Fig CADM1 and vFLIP were localized in the membrane lipid rafts. (A) BCBL-1 cells were stained with DAPI, anti-vFLIP, anti-CADM1, and cholera toxin B conjugated with red fluorescence to detect GM-1 and subjected to confocal microscopy. (B) BCBL-1 were treated with 10 mM MβCD for 30 min and stained with DAPI, anti-vFLIP, anti-CADM1, and cholera toxin B conjugated with red fluorescence to detect GM-1 and subjected to confocal microscopy.
(TIF)

S14 Fig Disruption of lipid rafts impairs CADM1, vFLIP and NEMO interactions and IKK complex activation. (A) BC-3 cells were treated with 10 mM MβCD for 30 min and stained with DAPI, anti-vFLIP, anti-CADM1, and cholera toxin B conjugated with red fluorescence to detect GM-1 and subjected to confocal microscopy. (B) Lipid raft fractionations of BC-3 cells pretreated with MβCD were subjected to immunoprecipitation with anti-vFLIP and immunoblotted with anti-vFLIP, anti-CADM1, and NEMO. Lysates from lipid rafts fractions were examined for vFLIP, phospho-IKKα/β, total IKKα, IKKβ, NEMO, CADM1, ERK1 (marker for soluble fractions), and Lyn (lipid raft protein marker).
(TIF)

S15 Fig Time course of cell viability, IκBα phosphorylation and degradation upon silencing of CADM1 expression in BC3 cells. Cell viability assays were performed at 0, 24, 48, 72, and 96 hours after BC-3 cells were transduced with lentiviruses expressing the indicated shRNAs. Relative cell viability (%) was expressed as a percentage relative to the control cells. The lysates were subjected to immunoblotting to examine CADM1, IκBα phosphorylation, IκBα degradation, and β-actin expression.
(TIF)

S16 Fig CADM1 does not affect the viability of BJAB cells. (A) Cell viability assay was performed 96 hours after BJAB cells were transduced with lentiviruses expressing the indicated shRNAs. Relative cell viability (%) was expressed as a percentage relative to the control cells. (B) CADM1 protein was knocked down in BJAB cells after lentiviral transduction expressing the indicated shRNAs. Immunoblotting was performed with whole cell lysates. (C) Flow cytometric analysis of BJAB cell lines transduced with shRNAs as described in (A). Cells were stained with both annexin-V-Alexa Fluor 488 and propidium iodide (PI). The distribution of cells is indicated as a percentage in each quadrant.
(TIF)

S17 Fig CADM1 is essential for the survival of BC-1 PEL cell line. BC-1 cells were transfected with 3’UTR CADM1 siRNA followed by transduction of lentiviruses containing wild-type flag-CADM1 as indicated. Cells were stained with both annexin-V-Alexa Fluor 488 and propidium iodide (PI). The distribution of cells is indicated as a percentage in each quadrant.
(TIF)

S18 Fig A schematic model of CADM1, vFLIP, and vGPCR-mediated NF-κB activation. Membrane associated CADM1 interacts with vFLIP and vGPCR, which leads to the activation of the IKK kinase complex and NF-κB and proinflammatory cytokine production.
(TIF)
