# Supplementary figures and images for: CADM1 is essential for KSHV-encoded vGPCR-and vFLIP-mediated chronic NF-κB activation
Source: PLoS Pathog. 2018 Apr 26;14(4):e1006968. doi: 10.1371/journal.ppat.1006968 (PMC5919438; doi:10.1371/journal.ppat.1006968)

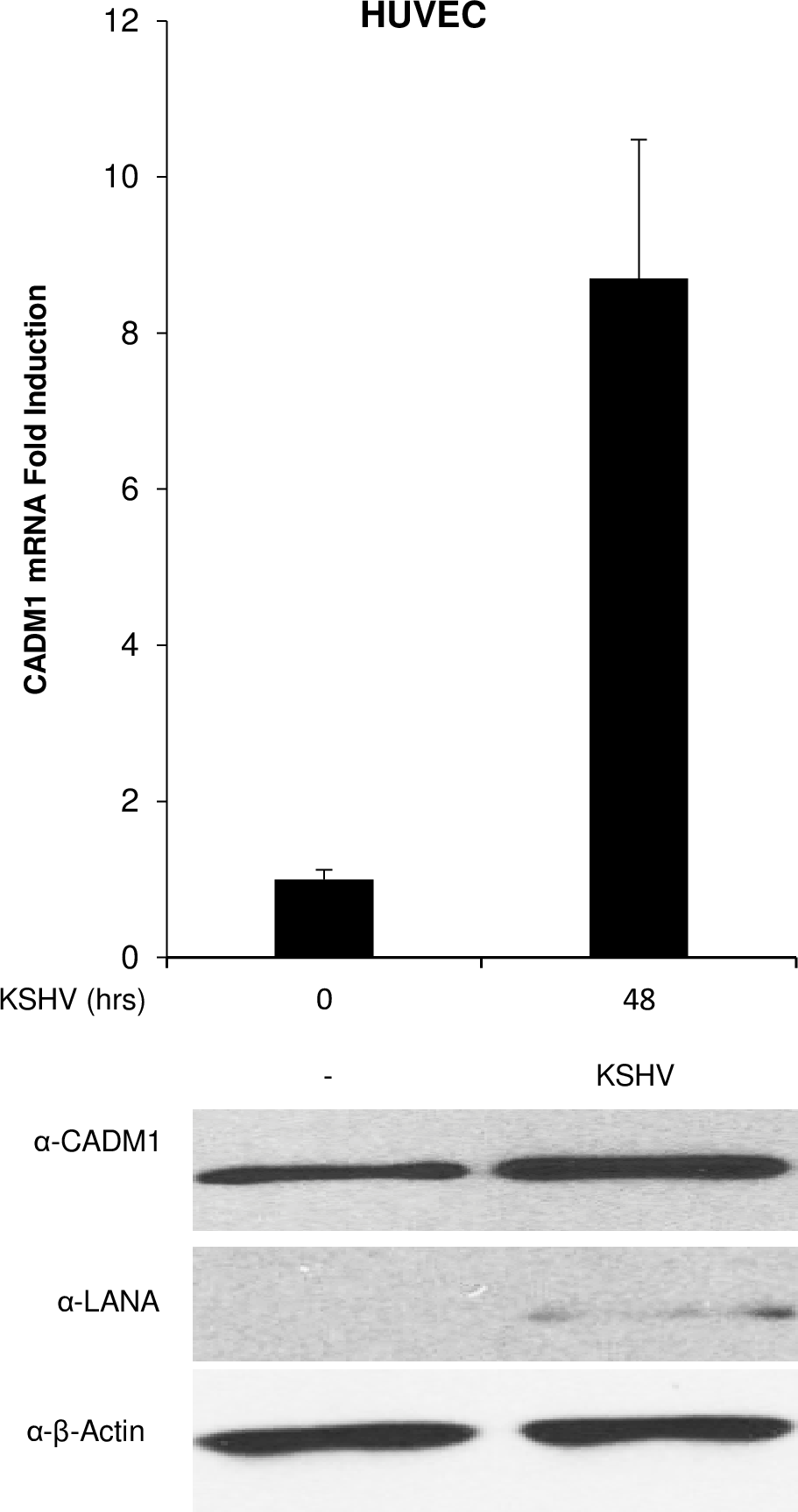

Supplement: S1 Fig — HUVEC cells were infected (0.1 MOI) with KSHV. After 48 hours, total RNA was prepared and subjected to quantitative PCR for CADM1 mRNA. The lysates were also subjected to immunoblotting to examine CADM1, KSHV-associated protein, LANA and β-actin expression. (TIF) [file ppat.1006968.s001.tif]

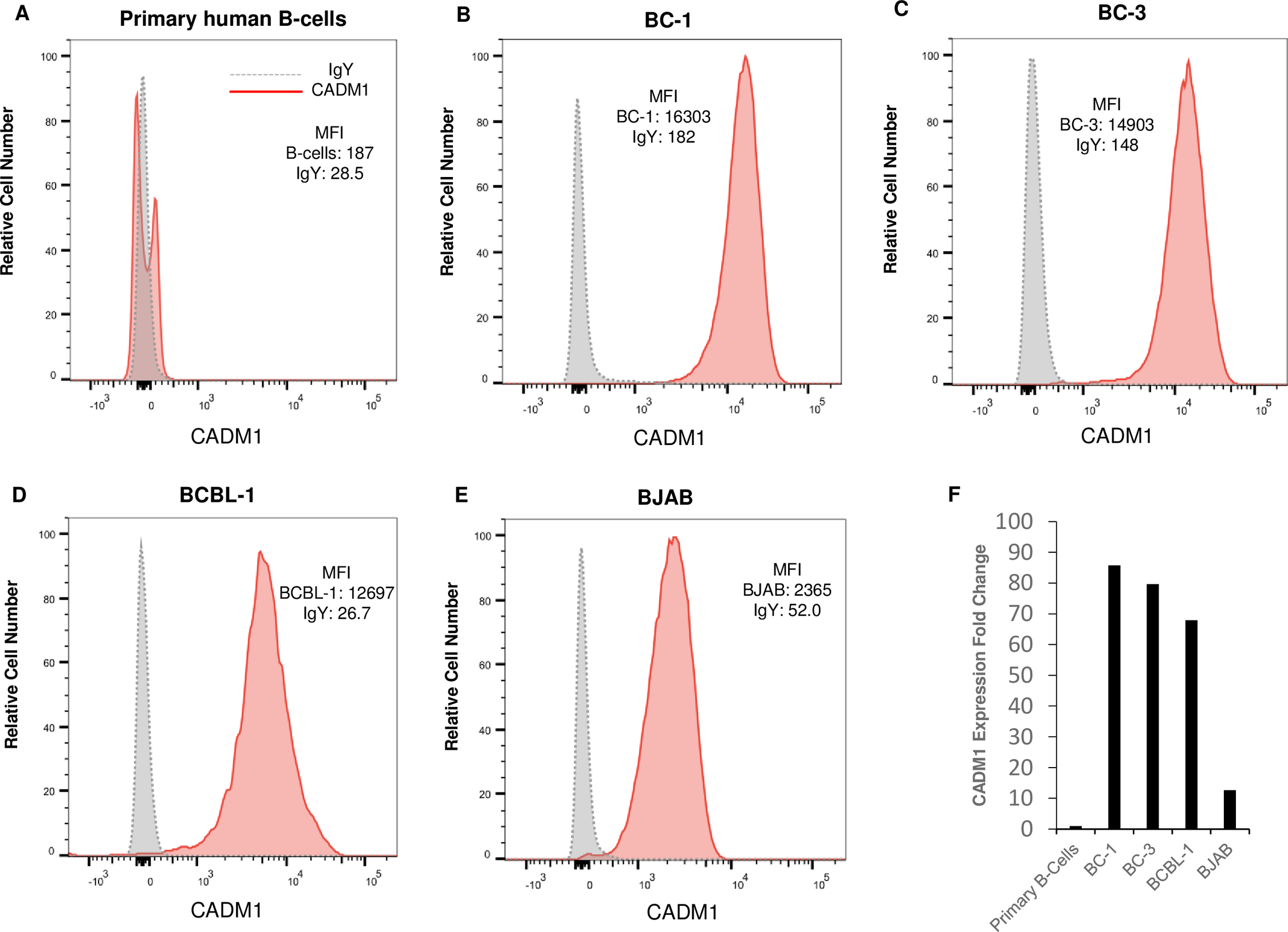

Supplement: S2 Fig — CADM1 expression was assessed in primary human B cells, PEL cell lines, and non-infected BJAB cell lines by flow cytometry. Representative histograms are shown. Black dotted lines correspond to IgY control and Red color histograms correspond to CADM1 expression in primary human B cells (A), PEL cells BC-1 (B), BC-3 (C), BCBL-1(D), and BJAB (E). (F) MFI of CADM1 expression in primary human B cells, BC-1, BC-3, BCBL-1 and BJAB, respectively. (TIF) [file ppat.1006968.s002.tif]

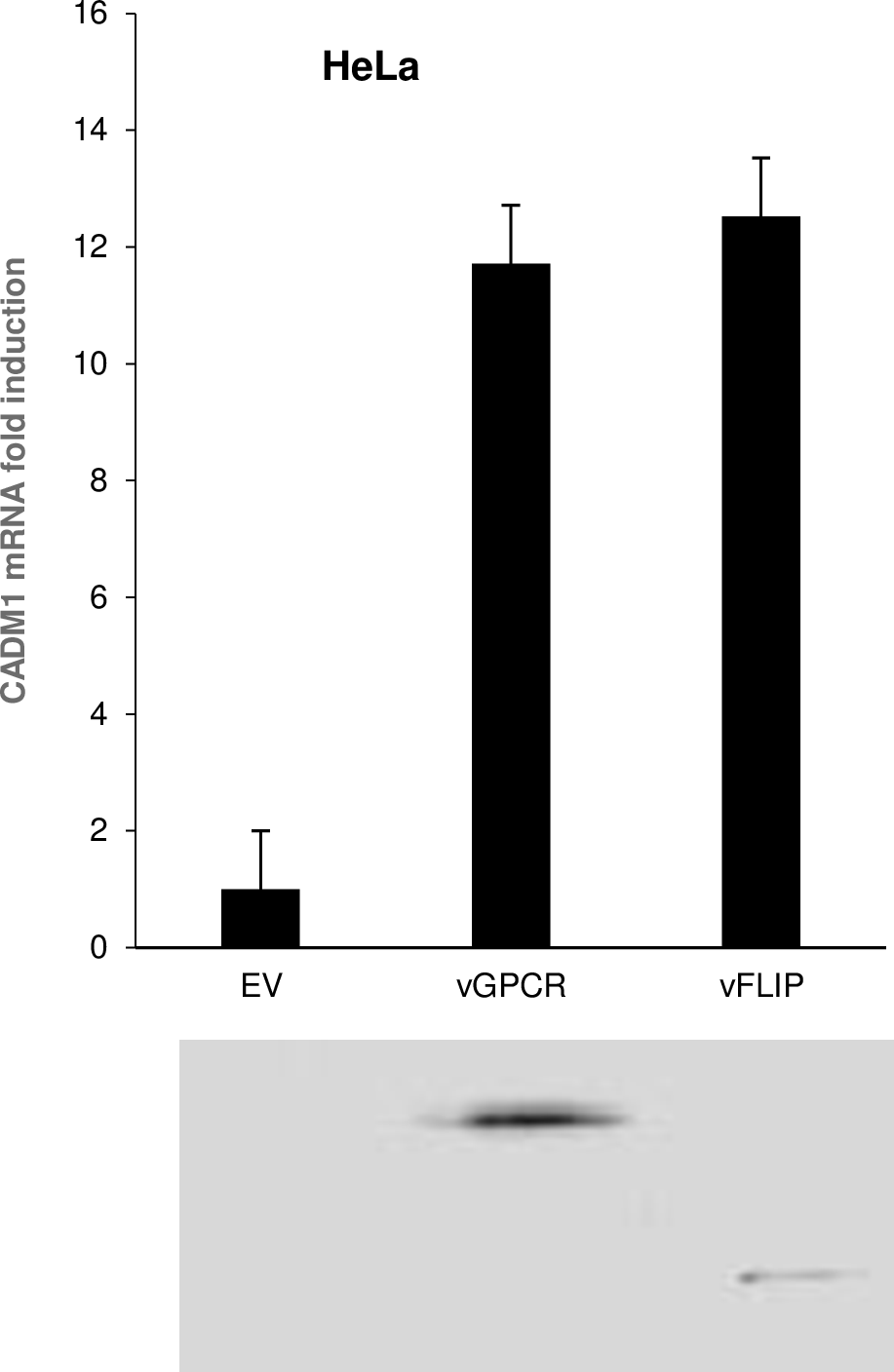

Supplement: S3 Fig — HeLa cells were transfected with vGPCR or vFLIP plasmids. After 48 hours, total RNA was prepared and subjected to quantitative PCR for CADM1 mRNA. The lysates were also subjected to immunoblotting to examine Flag-tagged, vGPCR and vFLIP expression. (TIF) [file ppat.1006968.s003.tif]

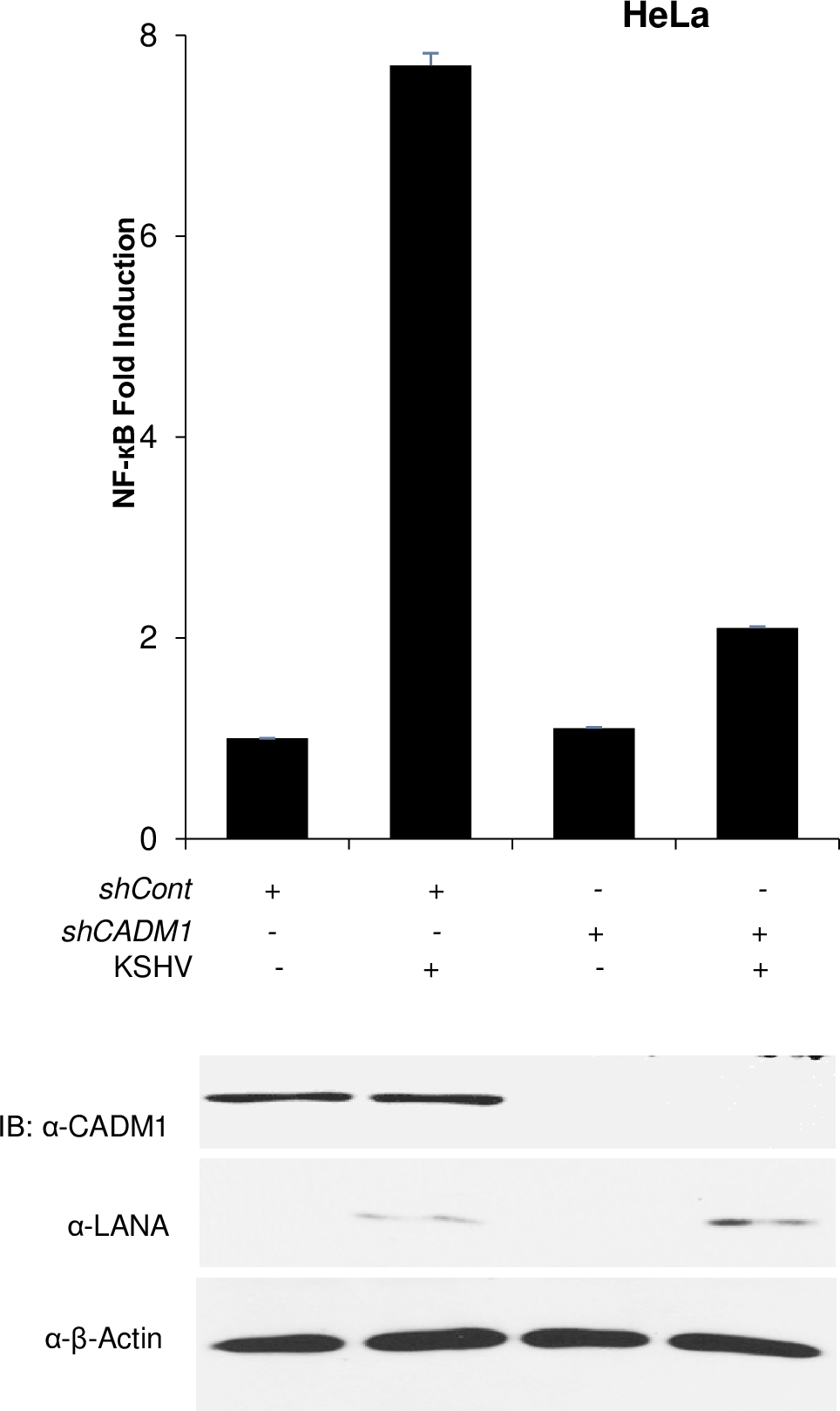

Supplement: S4 Fig — NF-κB luciferase assay using lysates of HeLa cells expressing control scrambled shRNA or CADM1 shRNA (+/- infection with KSHV (0.1 MOI)) and transfected with pRL-tk internal control Renilla luciferase plasmid, κB-TATA Luc for 24 hours as indicated. After 24 hours of infection, lysates were subjected to dual luciferase assays. The lysates were also subjected to immunoblotting to examine CADM1, KSHV-associated protein, LANA, and β-actin expression. (TIF) [file ppat.1006968.s004.tif]

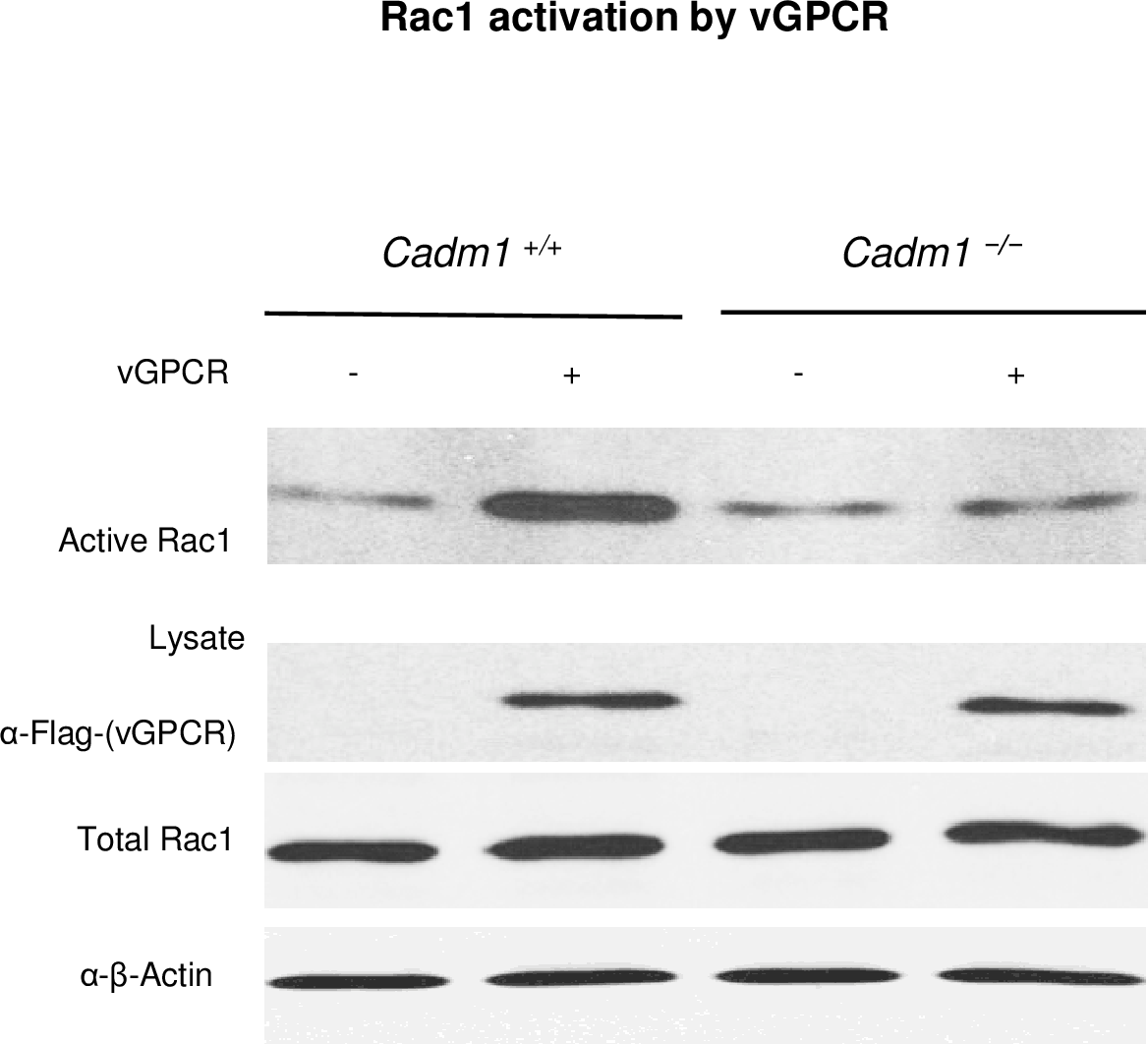

Supplement: S5 Fig — Equal amount of lysates of Cadm1 +/+ and Cadm1 −/− MEFs expressing vGPCR were incubated with PAK-PBD. Active Rac1, Flag-vGPCR expression, total Rac1, and β-actin were detected by western blotting. (TIF) [file ppat.1006968.s005.tif]

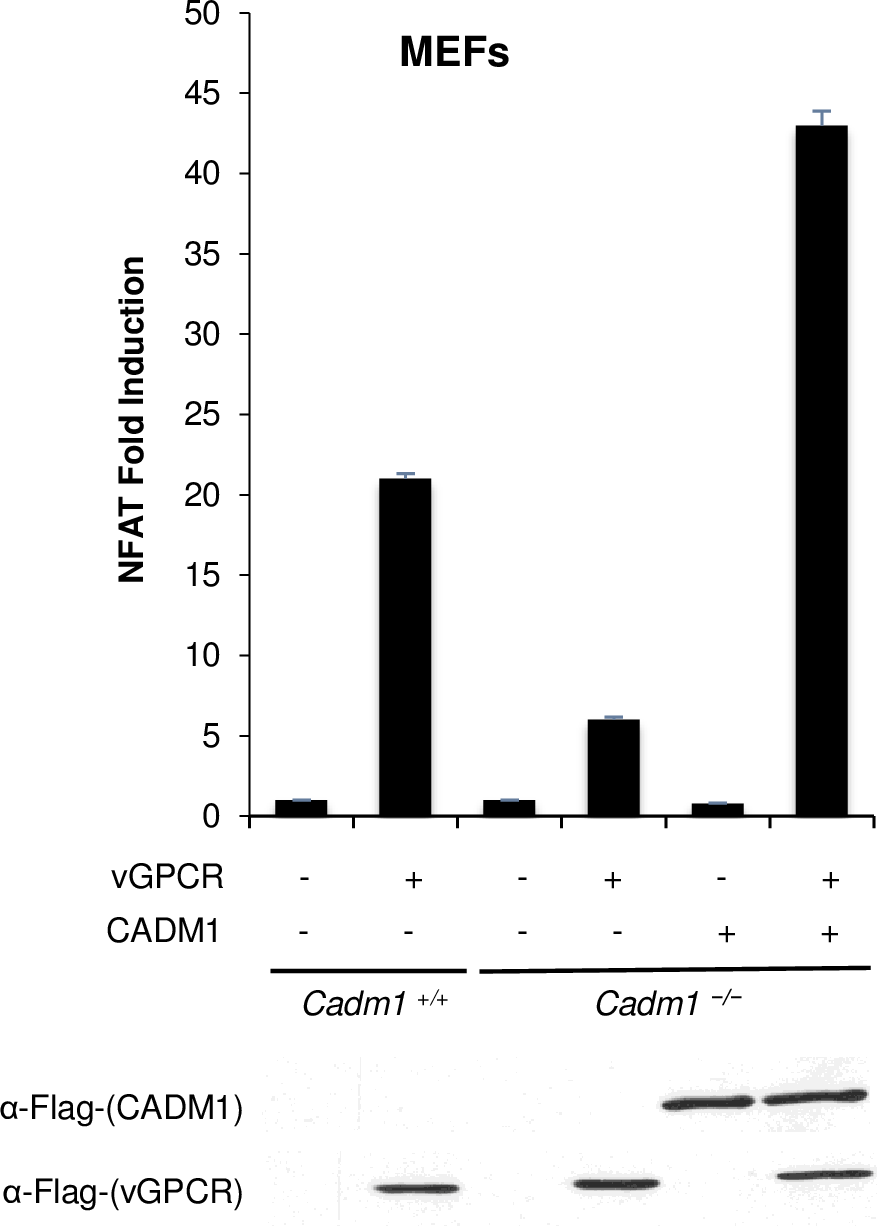

Supplement: S6 Fig — Cadm1+/+, Cadm1-/-, and Cadm1-/- MEFs reconstituted with wild-type Flag-tagged CADM1 were transfected with an NFAT-dependent luciferase reporter construct and vGPCR. After 36 hours, cells were lysed and subjected to immunoblotting to examine CADM1 and vGPCR expression using anti-Flag antibody. (TIF) [file ppat.1006968.s006.tif]

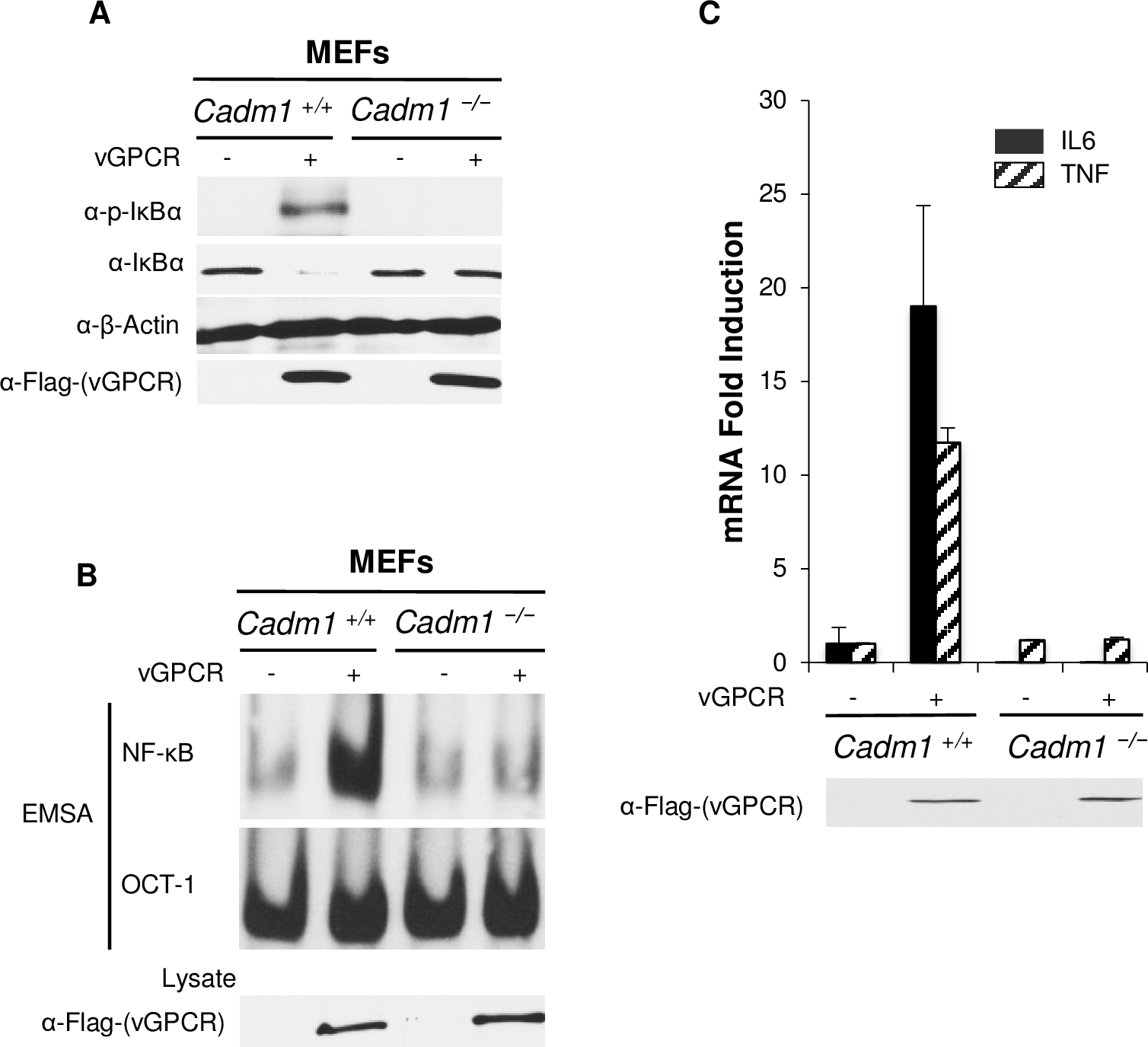

Supplement: S7 Fig — (A) Primary Cadm1+/+ and Cadm1−/− MEFs were transfected with vGPCR plasmid. After 48 h, lysates were subjected to immunoblotting with anti-phospho-IκBα, anti-CADM1, and anti-Flag antibodies. (B) Nuclear extracts from primary Cadm1+/+ and Cadm1−/− MEFs transfected with vGPCR were used for NF-κB and Oct-1 EMSA, and cytoplasmic extracts were subjected to immunoblotting with anti-Flag antibody. (C) Quantitative real-time PCR (qRT-PCR) analysis of Tnf and Il-6 from Cadm1+/+ and Cadm1−/− MEFs expressing vGPCR for 48 hours. Lysates were subjected to immunoblotting with anti-Flag for vGPCR protein expression. (TIF) [file ppat.1006968.s007.tif]

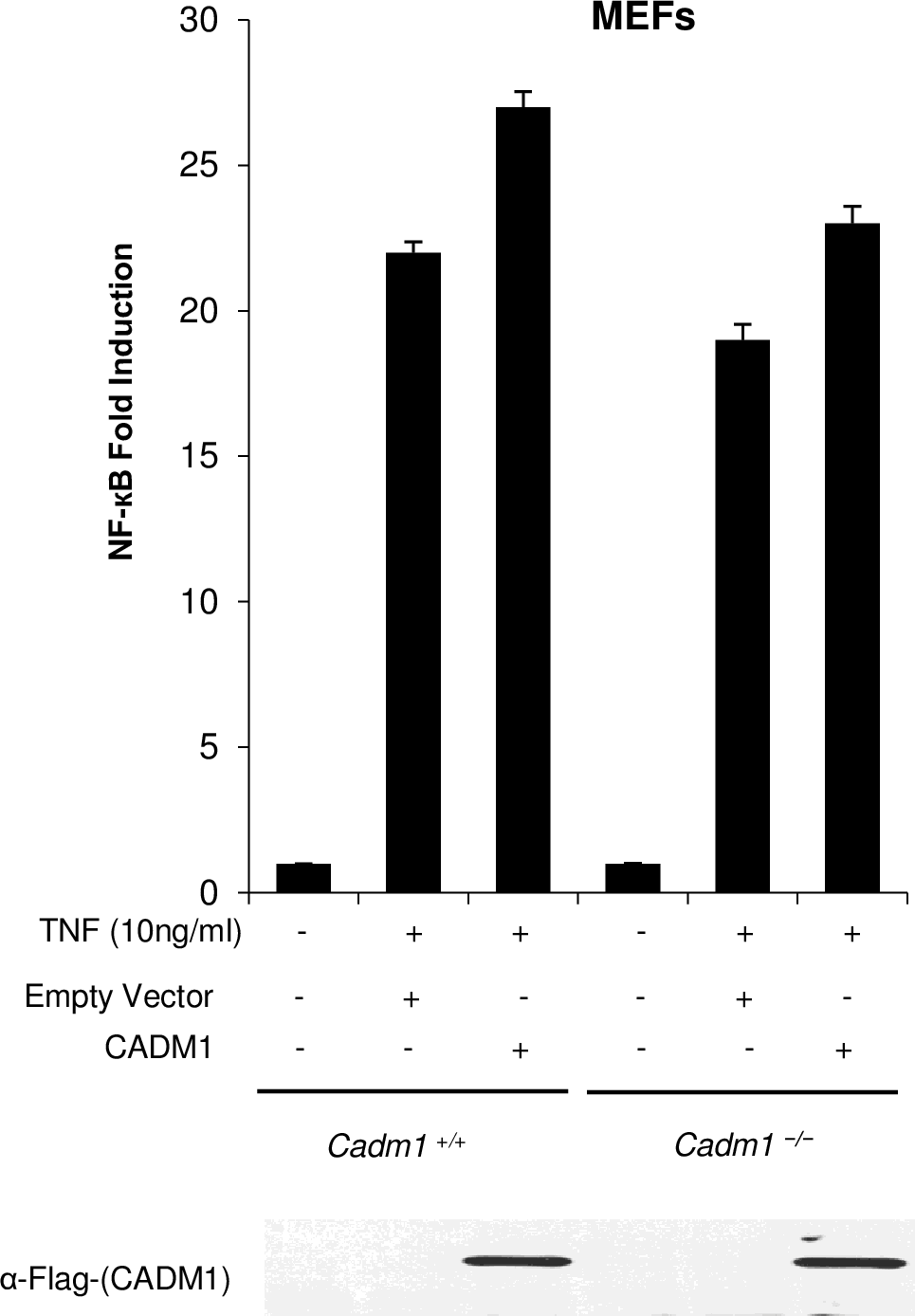

Supplement: S8 Fig — NF-κB luciferase assay using lysates of Cadm1+/+ and Cadm1-/- MEFs transfected with either empty vector, CADM1, and κB‐TATA Luc and pRL‐tk and stimulated with TNFα for 8 hours. Lysates were subjected to dual luciferase assays. The lysates were also subjected to immunoblotting to examine CADM1, expression using anti-Flag antibody. (TIF) [file ppat.1006968.s008.tif]

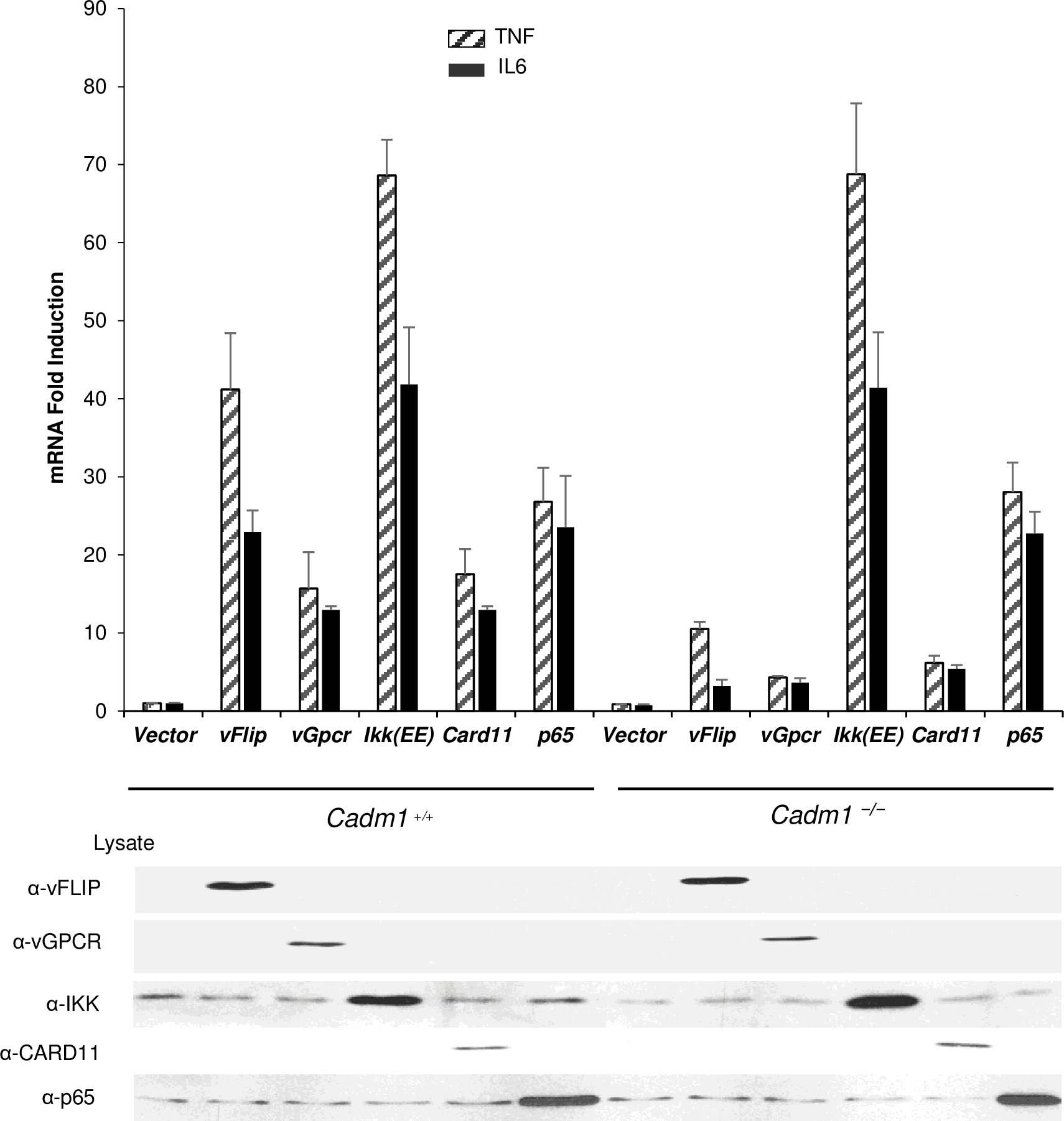

Supplement: S9 Fig — Cadm1 +/+ and Cadm1 −/− MEFs were transfected with either Empty Vector, vFLIP, vGPCR, IKK(EE), CARD11, or p65. After 36 hours, total RNA was prepared and subjected to quantitative PCR for Tnf and Il-6 mRNAs. The lysates were also subjected to immunoblotting to examine vFLIP, vGPCR, IKK, CARD11 and p65 expression using anti-Flag, anti-IKK, anti-Card11 and p65 antibodies, respectively. (TIF) [file ppat.1006968.s009.tif]

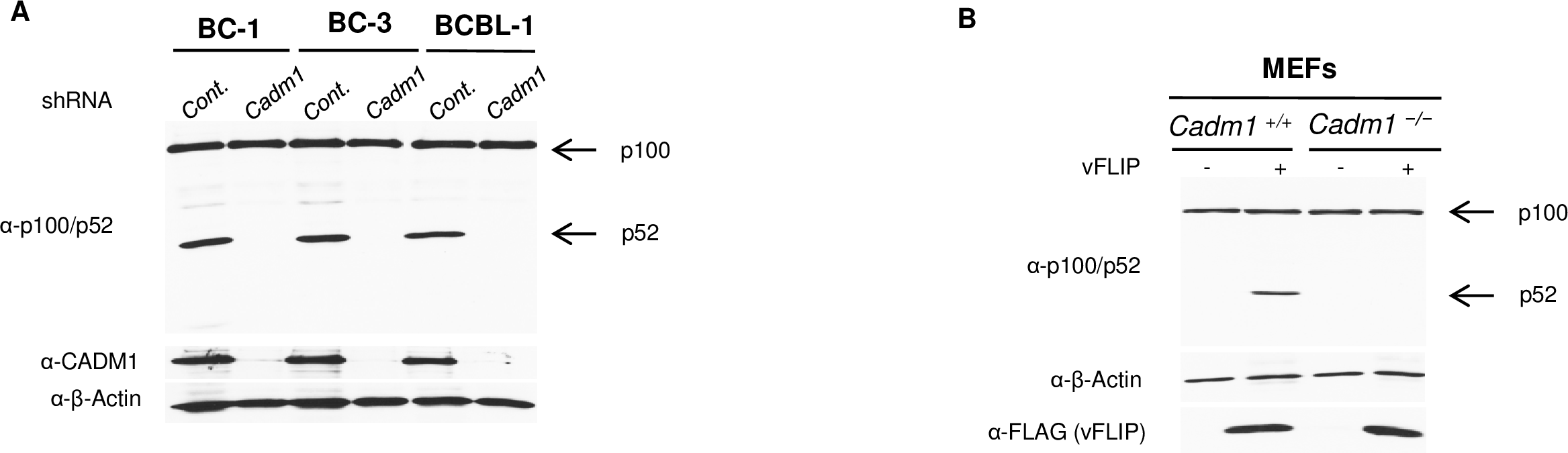

Supplement: S10 Fig — (A) Cell lysates from BC-1, BC-3, and BCBL-1 cells transduced with lentiviruses expressing the indicated shRNAs, were subjected to immunoblotting with anti-p100/p52, anti-CADM1, and anti-β-actin antibodies. (B) Lysates from primary Cadm1+/+ and Cadm1−/− MEFs transfected with vFLIP, immunoblotted with anti-Flag, anti-p100/p52, and anti-β-actin antibodies. (TIF) [file ppat.1006968.s010.tif]

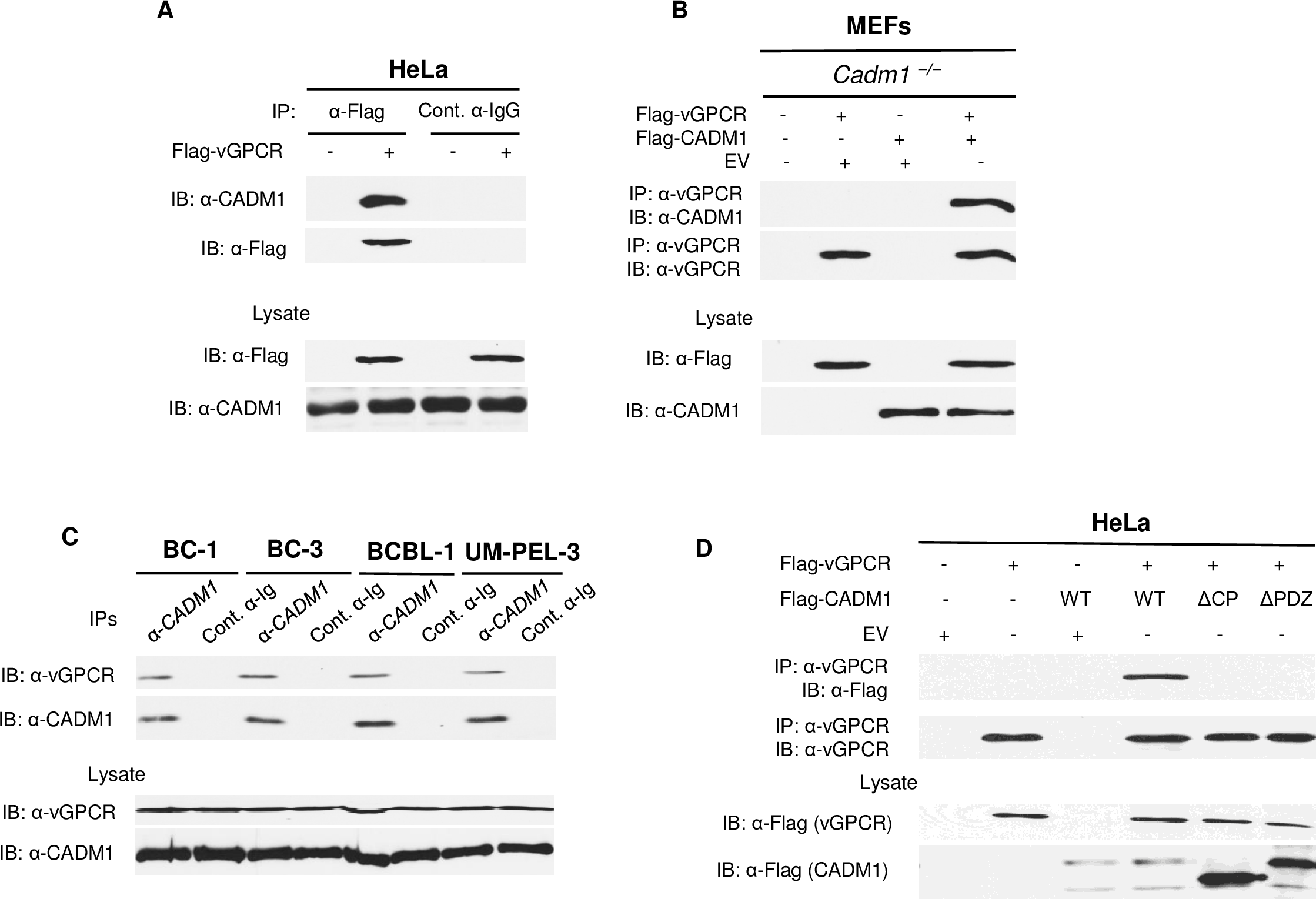

Supplement: S11 Fig — (A) HeLa cells were transfected with Flag-vGPCR. After 48 hours, cells were lysed and immunoprecipitated with either anti-Flag or control anti-IgG, followed by immunoblotting with anti-CADM1 and anti-Flag antibodies. Lysates were examined for Flag-vGPCR and CADM1 expression. (B) Primary Cadm1−/− MEFs were transfected with Flag-vGPCR expression vector, with or without Flag-CADM1. After 48 hours post-transfection, lysates were immunoprecipitated with anti-vGPCR and detected by immunoblotting with anti-CADM1 and vGPCR antibodies. Lysates were immunoblotted with anti-vGPCR, and anti-CADM1 antibodies. (C) Lysates from PEL cell lines (BC-1, BC-3, BCBL-1, and UM-PEL-3) were immunoprecipitated with either anti-CADM1 or control anti-IgG, followed by immunoblotting with anti-vGPCR and anti-CADM1. Lysates were examined for vGPCR and CADM1 expression. (D) Mapping the interaction between CADM1 and vGPCR. HeLa cells were transfected with vGPCR with the indicated Flag-CADM1 mutants. After 36 hours post-transfection, lysates were immunoprecipitated with anti-vGPCR and detected by immunoblotting with anti-Flag and anti-vGPCR antibodies. Lysates were immunoblotted with anti-Flag antibody. (TIF) [file ppat.1006968.s011.tif]

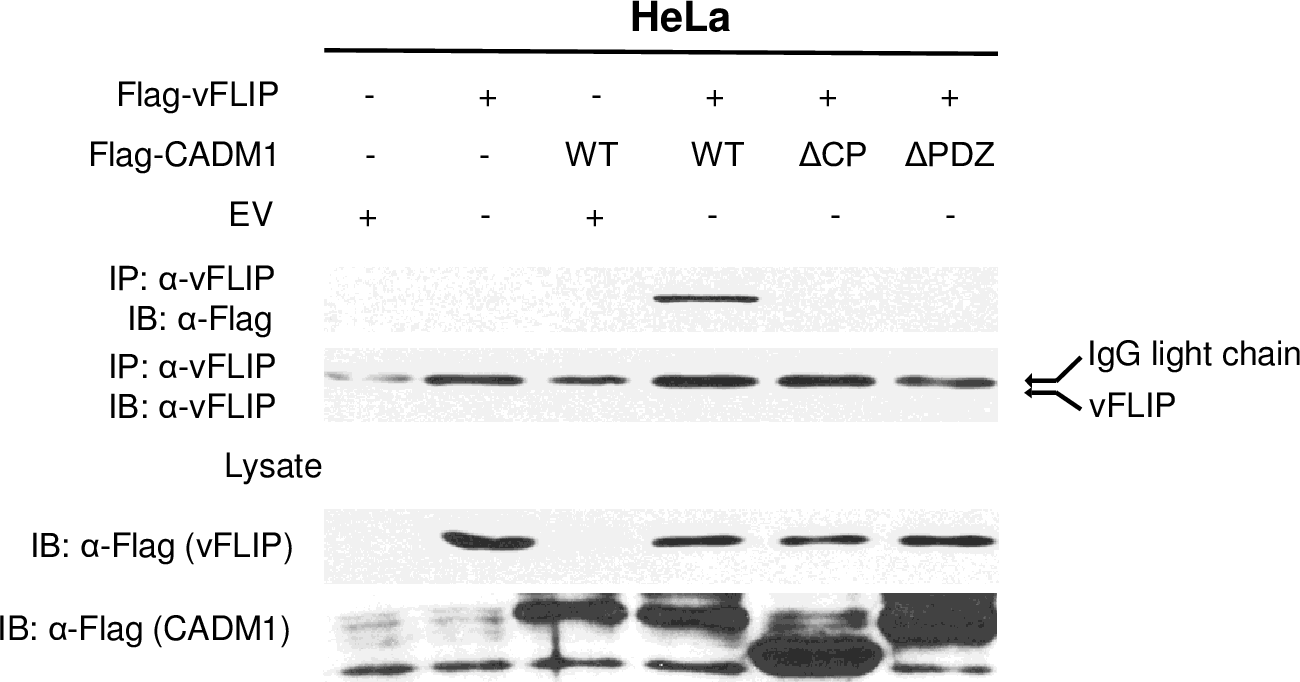

Supplement: S12 Fig — HeLa cells were transfected with a vFLIP expression vector together with the indicated Flag-CADM1 mutants. After 36 hours post-transfection, lysates were immunoprecipitated with anti-vFLIP and detected by immunoblotting with anti-Flag and anti-vFLIP antibodies. Lysates were immunoblotted with anti-Flag antibody. (TIF) [file ppat.1006968.s012.tif]

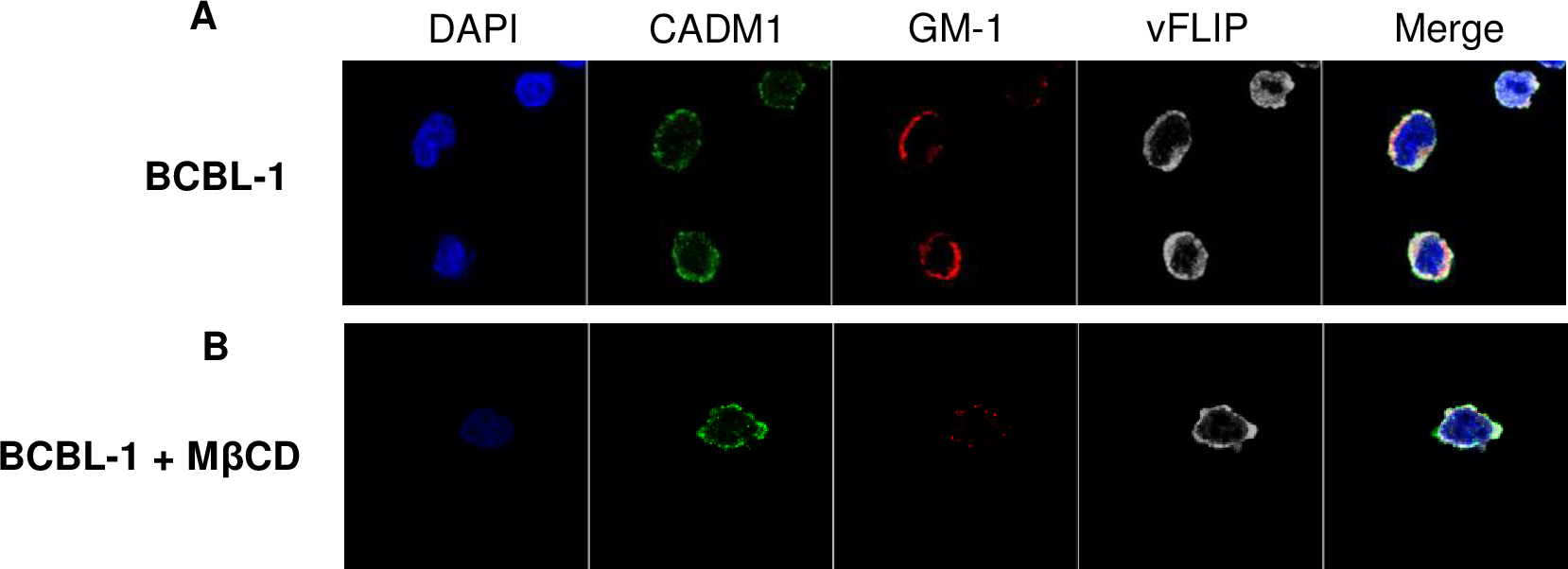

Supplement: S13 Fig — (A) BCBL-1 cells were stained with DAPI, anti-vFLIP, anti-CADM1, and cholera toxin B conjugated with red fluorescence to detect GM-1 and subjected to confocal microscopy. (B) BCBL-1 were treated with 10 mM MβCD for 30 min and stained with DAPI, anti-vFLIP, anti-CADM1, and cholera toxin B conjugated with red fluorescence to detect GM-1 and subjected to confocal microscopy. (TIF) [file ppat.1006968.s013.tif]

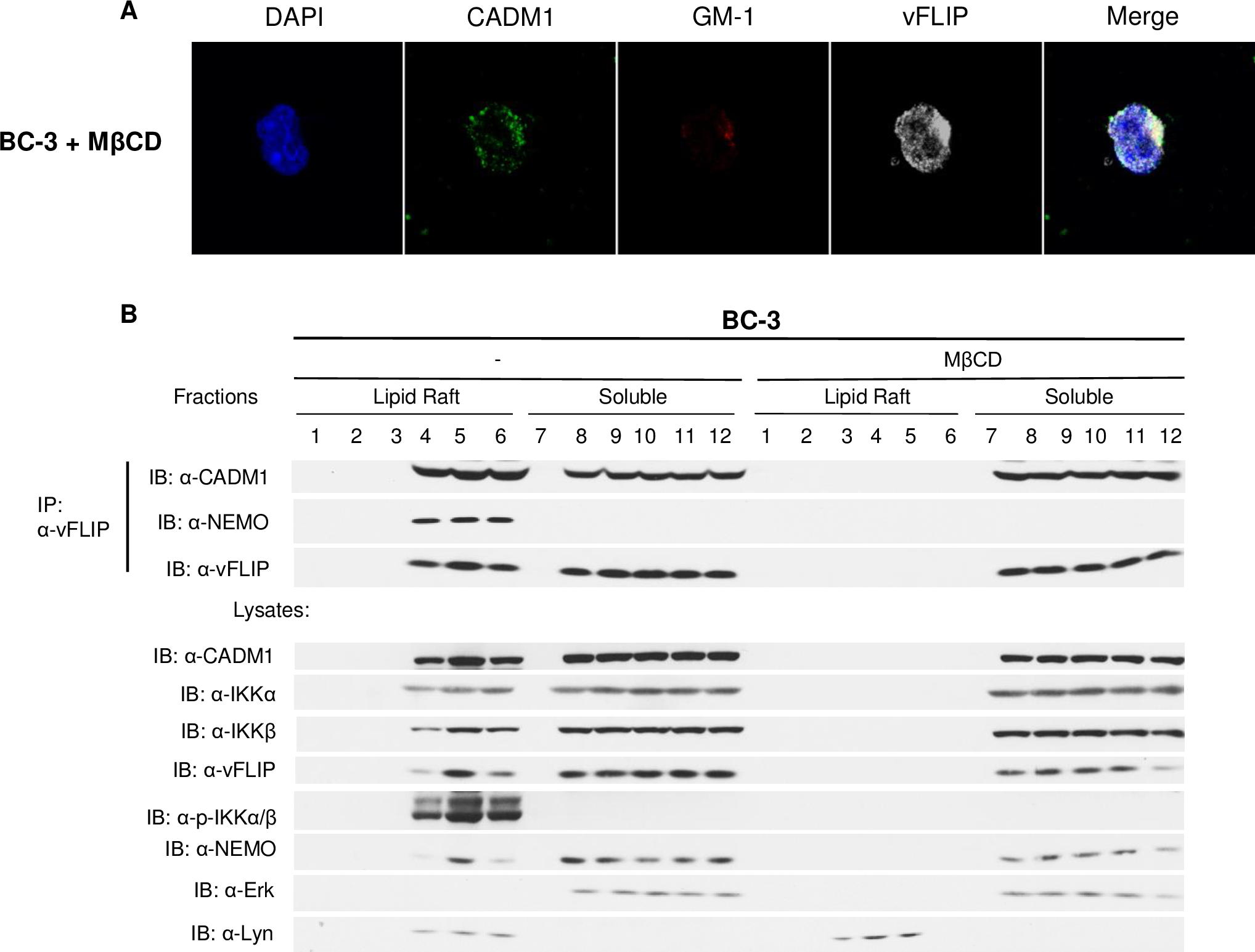

Supplement: S14 Fig — (A) BC-3 cells were treated with 10 mM MβCD for 30 min and stained with DAPI, anti-vFLIP, anti-CADM1, and cholera toxin B conjugated with red fluorescence to detect GM-1 and subjected to confocal microscopy. (B) Lipid raft fractionations of BC-3 cells pretreated with MβCD were subjected to immunoprecipitation with anti-vFLIP and immunoblotted with anti-vFLIP, anti-CADM1, and NEMO. Lysates from lipid rafts fractions were examined for vFLIP, phospho-IKKα/β, total IKKα, IKKβ, NEMO, CADM1, ERK1 (marker for soluble fractions), and Lyn (lipid raft protein marker). (TIF) [file ppat.1006968.s014.tif]

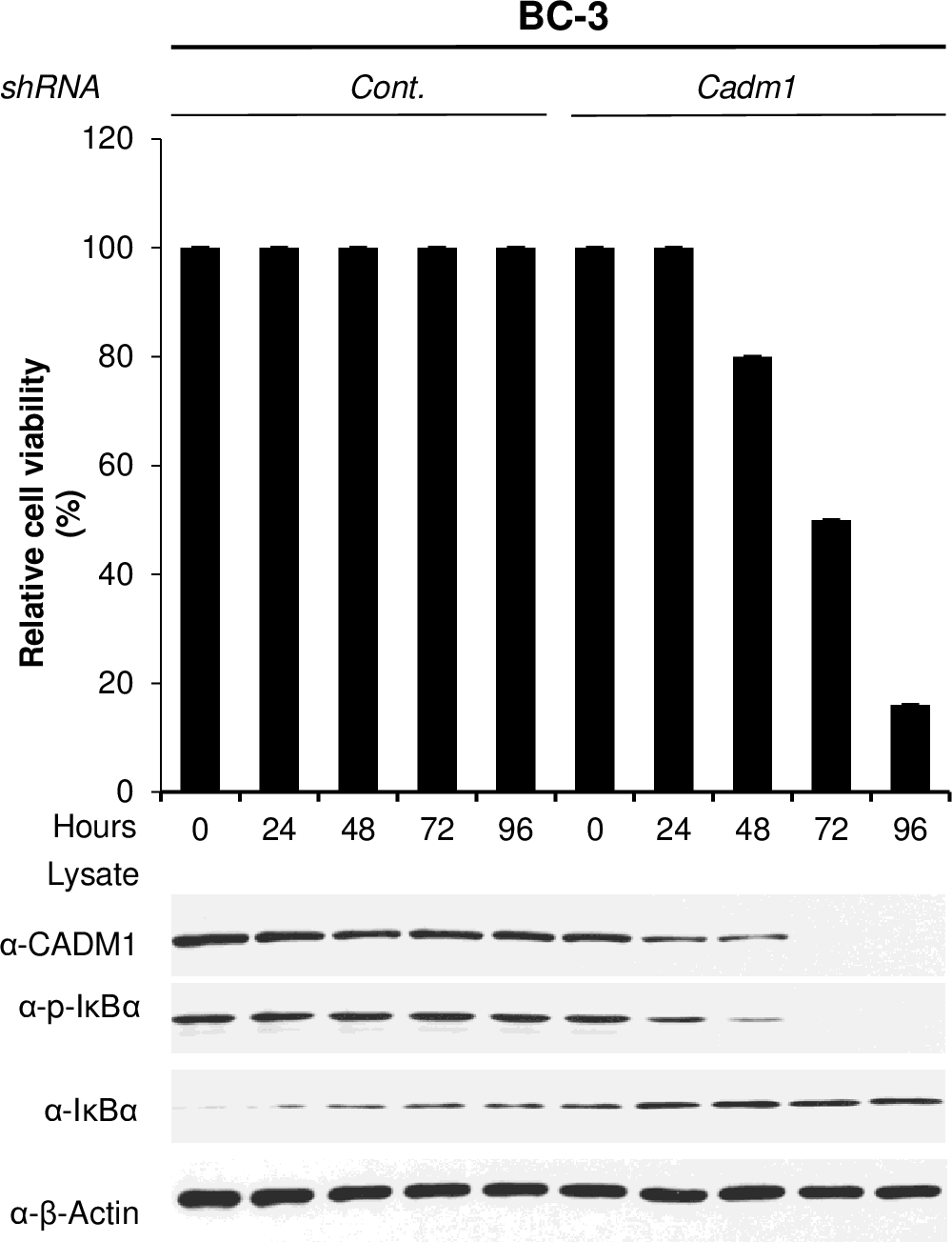

Supplement: S15 Fig — Cell viability assays were performed at 0, 24, 48, 72, and 96 hours after BC-3 cells were transduced with lentiviruses expressing the indicated shRNAs. Relative cell viability (%) was expressed as a percentage relative to the control cells. The lysates were subjected to immunoblotting to examine CADM1, IκBα phosphorylation, IκBα degradation, and β-actin expression. (TIF) [file ppat.1006968.s015.tif]

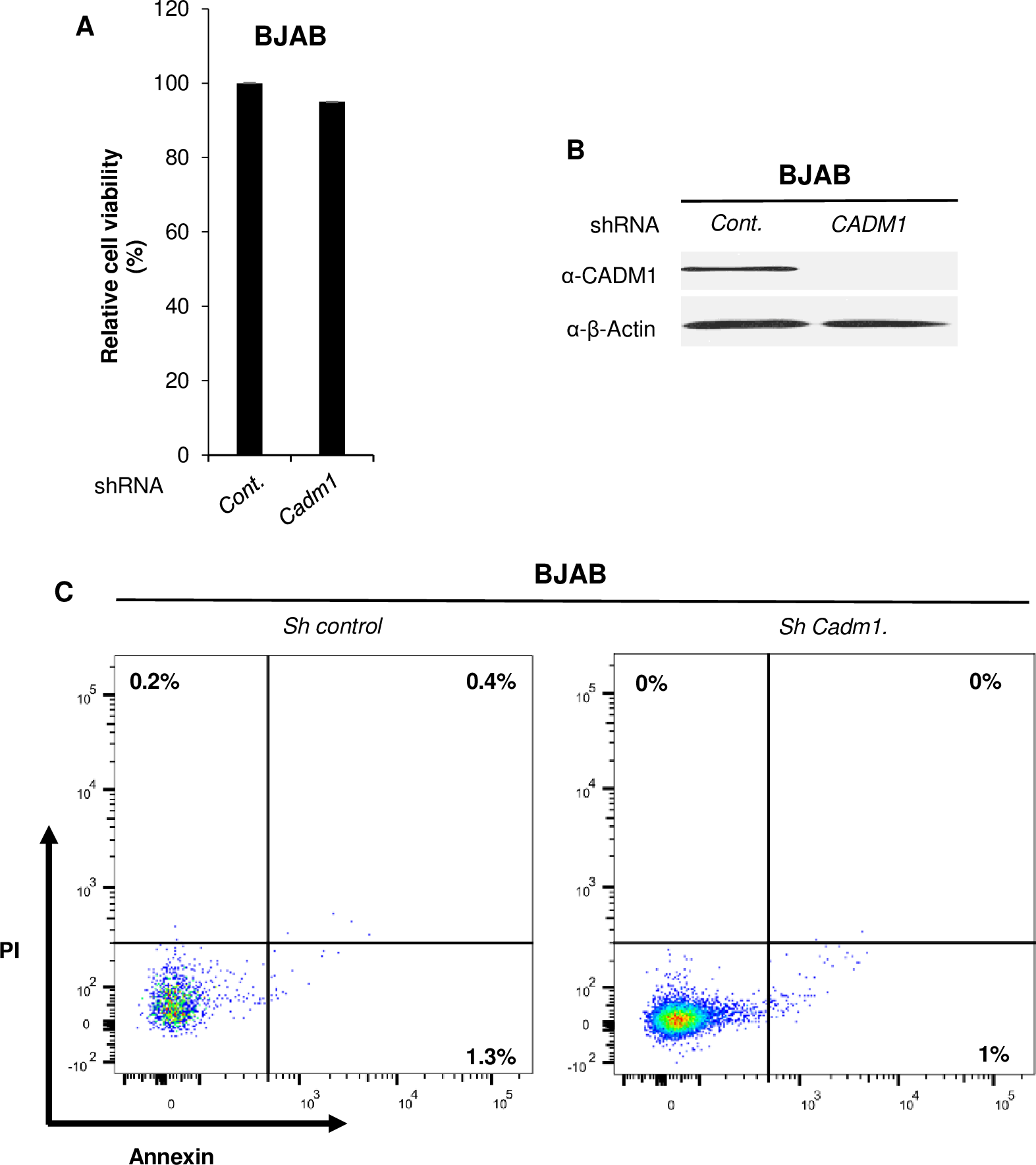

Supplement: S16 Fig — (A) Cell viability assay was performed 96 hours after BJAB cells were transduced with lentiviruses expressing the indicated shRNAs. Relative cell viability (%) was expressed as a percentage relative to the control cells. (B) CADM1 protein was knocked down in BJAB cells after lentiviral transduction expressing the indicated shRNAs. Immunoblotting was performed with whole cell lysates. (C) Flow cytometric analysis of BJAB cell lines transduced with shRNAs as described in (A). Cells were stained with both annexin-V-Alexa Fluor 488 and propidium iodide (PI). The distribution of cells is indicated as a percentage in each quadrant. (TIF) [file ppat.1006968.s016.tif]

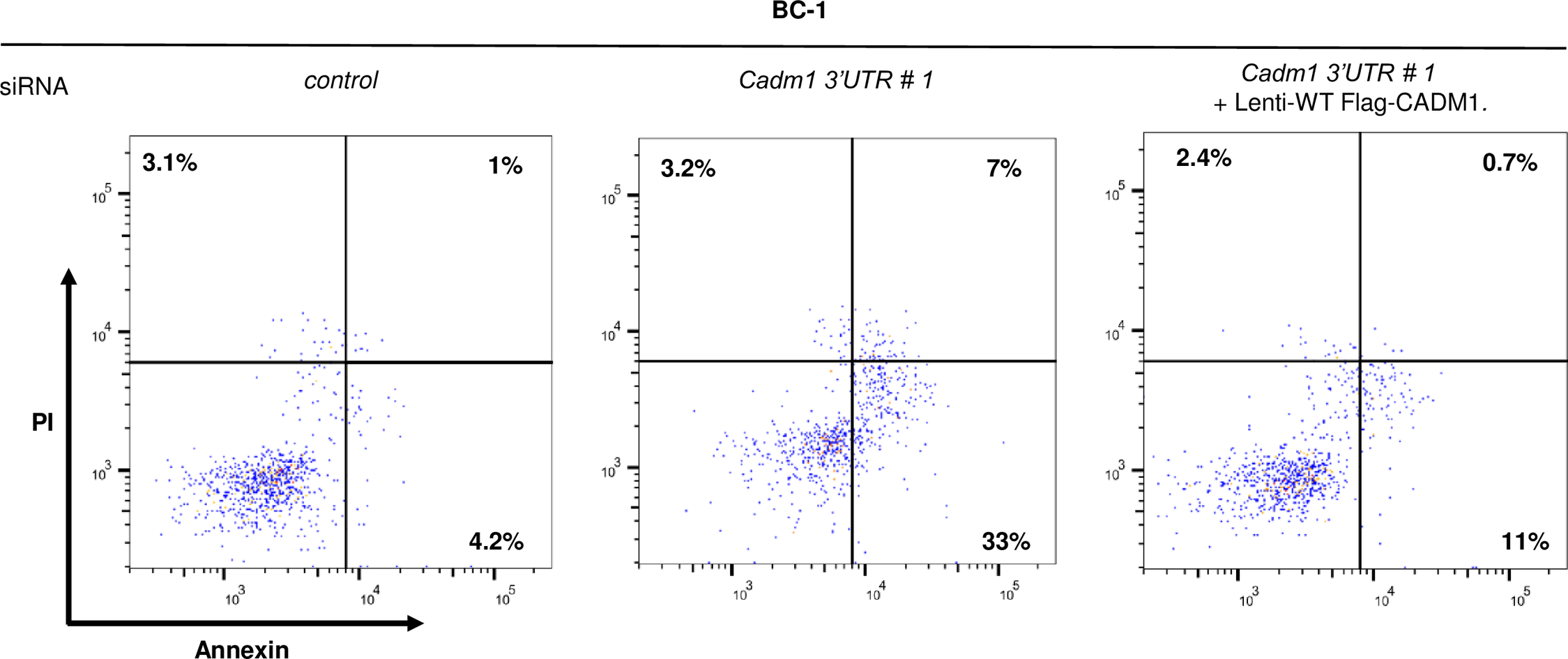

Supplement: S17 Fig — BC-1 cells were transfected with 3’UTR CADM1 siRNA followed by transduction of lentiviruses containing wild-type flag-CADM1 as indicated. Cells were stained with both annexin-V-Alexa Fluor 488 and propidium iodide (PI). The distribution of cells is indicated as a percentage in each quadrant. (TIF) [file ppat.1006968.s017.tif]

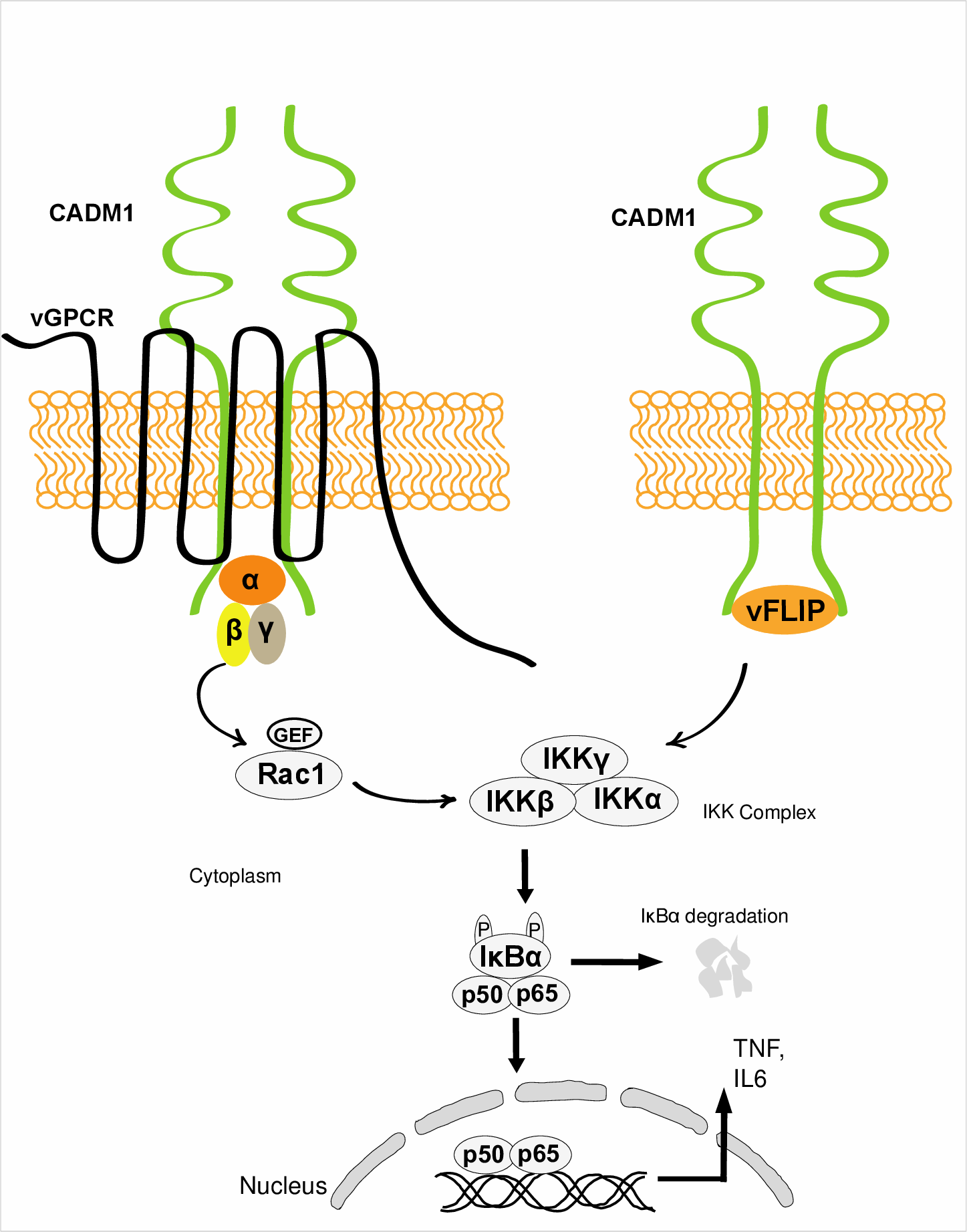

Supplement: S18 Fig — Membrane associated CADM1 interacts with vFLIP and vGPCR, which leads to the activation of the IKK kinase complex and NF-κB and proinflammatory cytokine production. (TIF) [file ppat.1006968.s018.tif]
